# Human Gray and White Matter Metabolomics to Differentiate APOE and Stage Dependent Changes in Alzheimer’s Disease

**DOI:** 10.33696/immunology.3.123

**Published:** 2021

**Authors:** Tyler C. Hammond, Xin Xing, Lucy M. Yanckello, Arnold Stromberg, Ya-Hsuan Chang, Peter T. Nelson, Ai-Ling Lin

**Affiliations:** 1 Sanders-Brown Center on Aging, University of Kentucky, Lexington, KY, USA; 2 Department of Neuroscience, University of Kentucky, Lexington, KY, USA; 3 Department of Computer Science, University of Kentucky, Lexington, KY, USA; 4 Department of Pharmacology and Nutritional Sciences, University of Kentucky, Lexington, KY, USA; 5 Department of Statistics, University of Kentucky, Lexington, KY, USA; 6 Department of Pathology, University of Kentucky, Lexington, KY, USA; 7 Department of Radiology and Biological Sciences, University of Missouri, Columbia, MO, USA

## Abstract

Alzheimer’s disease (AD) is the most common form of dementia with hallmarks of β-amyloid (Aβ) plaques, tau tangles, and neurodegeneration. Studies have shown that neurodegeneration components, especially brain metabolic deficits, are more predictable for AD severity than Aβ and tau. However, detailed knowledge of the biochemical composition of AD brain tissue vs. normal brain tissue remains unclear. In this study, we performed a metabolomics analysis on the brain tissue of 158 community-based older adults in the University of Kentucky AD Research Center brain bank to characterize the biochemical profiles of brains with and without AD based on white/gray matter type, apolipoprotein E genotype (ε3 vs ε4 variants), and disease stage (early vs late) as all these factors influence metabolic processes. We also used machine learning to rank the top metabolites separating controls and AD in gray and white matter. Compared with control samples, we found that glutamate and creatine metabolism were more critical for predicting AD in the gray matter, while glycine, fatty acid, pyrimidine, tricarboxylic acid (TCA) cycle, and phosphatidylcholine metabolism were more critical in the white matter. In ε4 carriers, metabolites associated with the TCA cycle and oxidative phosphorylation were prominent in advanced stages compared to the early stages. In ε3 carriers, metabolites related to oxidative DNA damage, changes in inhibitory neurotransmitters, and disruptions of neuronal membranes were prominent in advanced stages compared to the early stages. In early disease, ε4 carriers had metabolites related to poor kidney function and altered neuronal sterol metabolism compared to ε3 carriers, but there were few differences between genotypes in late disease. Our results indicate that metabolism plays a pivotal role in differentiating *APOE*- and stage-dependent changes in AD and may facilitate precision lifestyle and dietary interventions to mitigate AD risk in the early stages, especially for ε4 carriers.

## Introduction

Alzheimer’s disease (AD) is a leading cause of death and morbidity in the United States [1]. The hallmarks of AD are β-amyloid (Aβ) and tau. However, studies have indicated that metabolic dysfunction may play a more pivotal role in the progression of AD [2]. Glucose hypometabolism and mitochondrial dysfunction are well-known features of AD [2]. These irregularities are likely influenced by Apolipoprotein E (*APOE*) genotype, the most common genetic risk factor for AD. APOE is a lipid transport carrier with a direct impact on metabolism whose function is dependent on the structure of the protein variant (whether it is ε2, ε3, or ε4). Those carriers of the *APOE* ε4 allele have a two- to four-fold increased risk for developing AD [3,4].

AD progresses differently in the white matter than the gray matter. While most research has been focused on reporting changes in the gray matter of the cortex, many studies are now reporting changes in white matter tracts [5]. Metabolism is different in gray versus white matter due to the unique composition of each type (more lipid metabolism in the white matter [6] and more glucose metabolism in the gray matter [7]). There is also evidence to suggest that there is more glycolytic metabolism in the white matter and more oxidative metabolism in the gray matter [8]. Understanding the metabolic demands of the two different brain environments could give important clues about the progression of AD.

Detailed knowledge of the biochemical composition of AD brain tissue vs normal brain tissue will be vital in understanding the metabolic processes underlying AD. To our knowledge, only three reports have been published describing a brain metabolomics signature of AD. These reports demonstrated that the principal metabolites that classify AD in brain tissue include glycerophospholipids, spermidine, sphingolipids, and changes in bile acids [9–11]. These reports analyzed between 15 and 111 samples per group to develop a brain metabolomics signature based on targeted lipids or bile acids assays. The analyses were based on the gray-enriched matter only. While these reports provide an introductory study of brain metabolomics, a complete understanding is needed that factors a larger patient population, an untargeted assay, and samples enriched for both gray and white matter. Furthermore, a better understanding of how AD progresses based on *APOE* genotype is needed to develop therapeutics for AD based on precision medicine eventually.

Here we performed a metabolomics analysis on the brain tissue of a large cohort of community-based participants in the University of Kentucky Alzheimer’s Disease Research Center (UK-ADRC) brain bank. We used machine learning to identify the differences between the biochemical profiles of white-enriched matter and gray-enriched matter and identified the metabolites which were the top predictors in differentiating AD from normal brain tissue. We proceeded to characterize the biochemical profiles of brains with and without AD based on *APOE* genotype and disease stage.

## Methods

### Participant characteristics

Details of UK-ADRC research volunteers’ recruitment, inclusion/exclusion criteria, and clinical and pathological assessments have been described previously [12]. Briefly, community-based older adult volunteers agreed to be followed annually for cognitive, physical, and neurological examination and to donate their brains at the time of death. The UK Institutional Review Board approved protocols, and all participants provided written informed consent. Included subjects were ≥ 70 years of age at death. Research subjects with relatively rare dementia syndromes, e.g., prions, trinucleotide repeat diseases, or Frontotemporal lobar degeneration (FTLD), or any brain tumor were excluded.

Additionally, since our research questions focused on the association between *APOE* and tau pathology, cases also had to have *APOE* genotyping available and were dichotomized into *APOE* ε4 carriers and *APOE* ε3 homozygotes. Demographic information included our participants’ age at death (years), sex, race (nonwhite or white), and years of education. We describe diagnosed cognitive status (normal cognition, impaired not mild cognitive impairment [MCI], MCI, dementia), and primary clinical diagnosis (normal cognition, AD, LBD/Parkinson disease; vascular disease, other) at the participant’s last visit before death. Using Braak NFT staging [13], we defined those as the early stage with a Braak NFT score 0-III and those as the late stage with a Braak score IV-VI. Participant characteristics are listed in Table 1.

**Figure 1:**
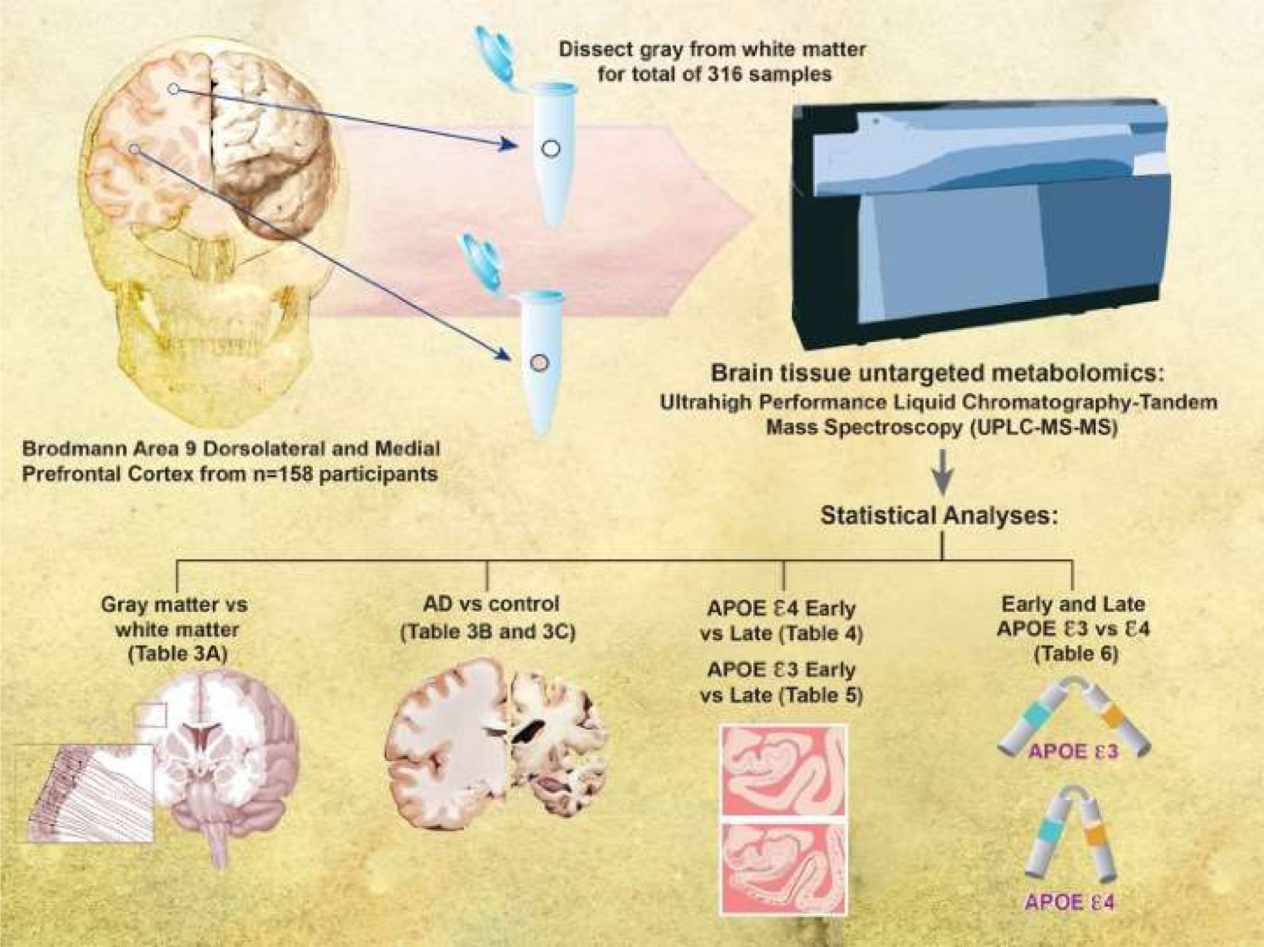
Schematic representation of study design. We took brain tissue from Brodmann Area 9 of 158 participants in the University of Kentucky Alzheimer’s Disease Center brain bank. We divided the samples into gray matter and white matter and performed untargeted metabolomics. We compared 1) Gray matter vs White matter, 2) AD vs control, 3) APOE 4 Early vs Late, 4) APOE 3 Early vs Late, and 5) Early and Late APOE 3 vs 4.

Figure 1 shows the schematic representation of the study design. Human brain tissue samples were collected and snap-frozen in liquid nitrogen at brain autopsy. For the present study, samples were obtained from Brodmann’s area 9 (the dorsolateral prefrontal cortex) of the left hemisphere of 158 community-based older adult volunteers. Brain tissue samples were further dissected into the gray-enriched matter and white-enriched matter samples, yielding 316 brain samples. The education level was the same across stage and genotype. Age differed between only late ε3 and late ε4 groups (87.97 ± 6.96 vs. 82.76 ± 8.45). We profiled the brain tissue metabolomics using Metabolon’s (Durham, NC) global screening platform. We separated our analyses by Braak NFT stages (early 0-III vs. late IV-VI) and *APOE* genotype (ε3 and ε4) (Table 2).

### Metabolon platform

We sent samples to Metabolon (Durham, NC) for a global metabolic profile for each sample. All samples were maintained at −80°C until processed. Samples were prepared using the automated MicroLab STAR® system from Hamilton Company. Proteins were precipitated with methanol under vigorous shaking for 2 min (Glen Mills GenoGrinder 2000) followed by centrifugation. The resulting extract was divided into five fractions: two for analysis by two separate reverse phases (RP)/UPLC-MS/MS methods with positive ion mode electrospray ionization (ESI), one for analysis by RP/UPLC-MS/MS with negative ion mode ESI, one for analysis by HILIC/UPLC-MS/MS with negative ion mode ESI, and one sample was reserved for backup. Samples were placed briefly on a TurboVap® (Zymark) to remove the organic solvent.

#### Ultrahigh performance liquid chromatography-tandem mass spectroscopy (UPLC-MS/MS):

All methods utilized a Waters ACQUITY ultra-performance liquid chromatography (UPLC) and a Thermo Scientific Q-Exactive high resolution/accurate mass spectrometer interfaced with a heated electrospray ionization (HESI-II) source and Orbitrap mass analyzer operated at 35,000 mass resolution. The sample extract was dried then reconstituted in solvents compatible with each of the four methods. Each reconstitution solvent contained a series of standards at fixed concentrations to ensure injection and chromatographic consistency. One aliquot was analyzed using acidic positive ion conditions, chromatographically optimized for more hydrophilic compounds. In this method, the extract was gradient eluted from a C18 column (Waters UPLC BEH C18–2.1×100 mm, 1.7 μm) using water and methanol, containing 0.05% perfluoropentanoic acid (PFPA) and 0.1% formic acid (FA). Another aliquot was also analyzed using acidic positive ion conditions; however, it was chromatographically optimized for more hydrophobic compounds. In this method, the extract was gradient eluted from the aforementioned C18 column using methanol, acetonitrile, water, 0.05% PFPA, and 0.01% FA and was operated at an overall higher organic content. Another aliquot was analyzed using basic negative ion optimized conditions using a separate dedicated C18 column. However, the essential extracts were gradient eluted from the column using methanol and water with 6.5mM Ammonium Bicarbonate at pH 8. The fourth aliquot was analyzed via negative ionization following elution from a HILIC column (Waters UPLC BEH Amide 2.1×150 mm, 1.7 μm) using a gradient consisting of water and acetonitrile with 10mM Ammonium Formate, pH 10.8. The MS analysis alternated between MS and data-dependent MS^n^ scans using dynamic exclusion. The scan range varied slightly between methods but covered 70–1000 m/z.

#### Bioinformatics:

The informatics system consisted of four major components, the Laboratory Information Management System (LIMS), the data extraction and peak-identification software, data processing tools for QC and compound identification, and a collection of information interpretation and visualization tools for use by data analysts. The hardware and software foundations for these informatics components were the LAN backbone, and a database server running Oracle 10.2.0.1 Enterprise Edition.

#### LIMS:

The purpose of the Metabolon LIMS system was to enable fully auditable laboratory automation through a secure, easy-to-use, and highly specialized system. The scope of the Metabolon LIMS system encompasses sample accessioning, sample preparation and instrumental analysis, and reporting an advanced data analysis. All the subsequent software systems are grounded in the LIMS data structures. It has been modified to leverage and interface with the in-house information extraction and data visualization systems, as well as third-party instrumentation and data analysis software.

#### Data extraction and compound identification:

Raw data was extracted, peak-identified, and QC processed using Metabolon’s hardware and software. These systems are built on a web-service platform utilizing Microsoft’s .NET technologies, which run on high-performance application servers and fiber-channel storage arrays in clusters to provide active failover and load-balancing. Compounds were identified by comparison to library entries of purified standards or recurrent unknown entities. Metabolon maintains a library based on authenticated standards that contain the retention time/index (RI), mass to charge ratio (*m/z)*, and chromatographic data (including MS/MS spectral data) on all molecules present in the library. Furthermore, biochemical identifications are based on three criteria: retention index within a narrow RI window of the proposed identification, accurate mass match to the library ± 10 ppm, and the MS/MS forward and reverse scores between the experimental data and authentic standards. The MS/MS scores are based on a comparison of the ions present in the experimental spectrum to the ions present in the library spectrum. While there may be similarities between these molecules based on one of these factors, all three data points can be utilized to distinguish and differentiate biochemicals. More than 3300 commercially available purified standard compounds have been acquired and registered into LIMS for analysis on all platforms to determine their analytical characteristics. Additional mass spectral entries have been created for structurally unnamed biochemicals, identified by virtue of their recurrent nature (both chromatographic and mass spectral). These compounds can be identified by the future acquisition of a matching purified standard or by classical structural analysis.

#### Metabolite quantification and data normalization:

**Figure 2:**
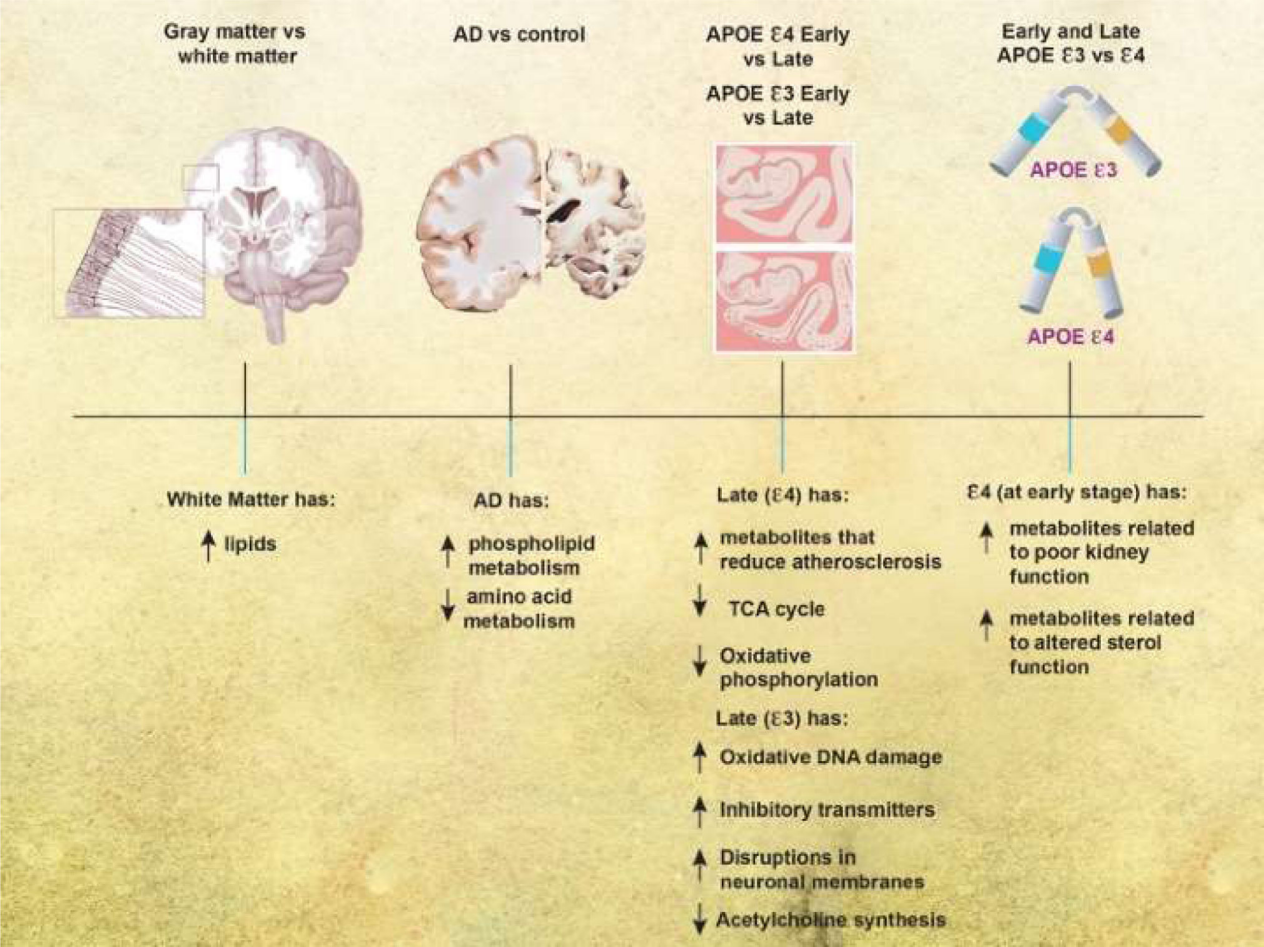
Schematic representation of overall results. Overall results from analysis of matter type, disease diagnosis, Braak stage, and APOE genotype.

Peaks were quantified using area-under-the-curve. For studies spanning multiple days, a data normalization step was performed to correct variation resulting from instrument inter-day tuning differences. Essentially, each compound was corrected in run-day blocks by registering the medians to equal one (1.00) and normalizing each data point proportionately (termed the “block correction”; Figure 2). For studies that did not require more than one day of analysis, no normalization is necessary, other than for data visualization purposes. In certain instances, biochemical data may have been normalized to an additional factor (e.g., cell counts, total protein as determined by Bradford assay, osmolality, etc.) to account for differences in metabolite levels due to differences in the amount of material present in each sample.

### Statistical and analytical methods

#### Statistical calculations:

For many studies, two types of statistical analysis are usually performed: (1) significance tests and (2) classification analysis. Standard statistical analyses are performed in ArrayStudio on log transformed data. For those analyses not standard in ArrayStudio, the programs R (http://cran.r-project.org/) or JMP are used. Below are examples of frequently employed significance tests and classification methods followed by a discussion of p- and q-value significance thresholds.

#### Machine learning classification:

Random Forest was used as a supervised classification technique to identify the relative importance of the different biochemicals in predicting gray-enriched matter vs. white-enriched matter, Alzheimer’s disease vs. control, *APOE* ε4 vs. *APOE* ε3 genotypes in early stage and late stage, early stage vs. late stage in *APOE* ε4 and *APOE* ε3 genotypes, and Vascular dementia vs. control based on an ensemble of decision trees. For a given decision tree, a random subset of the data with identifying true class information was selected to build the tree, and the remaining data, the “out-of-bag” (OOB) variables, were passed down the tree to obtain a class prediction for each sample. This process was repeated thousands of times to produce the forest. The final classification of each sample was determined by computing the class prediction frequency (“votes”) for the OOB variables over the whole forest. This method is unbiased since the prediction for each sample is based on trees built from a subset of samples that do not include that sample. When the full forest is grown, the class predictions are compared to the true classes, generating the “OOB error rate” to measure prediction accuracy. Thus, the prediction accuracy is an unbiased estimate of how well one can predict sample class in a new data set. The random forest has several advantages – it makes no parametric assumptions, variable selection is not needed, does not overfit, is invariant to transformation, and is relatively easy to implement with R.

To determine which variables (biochemicals) make the largest contribution to the classification, a “variable importance” measure is computed. We use the “Mean Decrease Accuracy” (MDA) as this metric. The MDA is determined by randomly permuting a variable, running the observed values through the trees, and then reassessing the prediction accuracy. If a variable is not important, then this procedure will have little change in the class prediction accuracy (permuting random noise will give random noise). By contrast, if a variable is important to the classification, the prediction accuracy will drop after such a permutation, which we record as the MDA. Thus, the random forest analysis provides an “importance” rank ordering of biochemicals; we typically output the top 30 biochemicals in the list as potentially worthy of further investigation.

## Results

### Metabolomics differences in gray and white-enriched matters

In total, 540 of the 776 detected metabolites were either increased or decreased in the white-enriched matter vs gray-enriched matter. The white-enriched matter has more lipid metabolites associated with myelin than gray-enriched matter. A detailed list is found in Supplementary Table 1.

Using a random forest analysis, we found that the model could predict whether a sample was the gray-enriched matter or white-enriched matter based on the metabolomics profile with 91.77% accuracy (Table 3A). Heat map of statistically significant biochemicals profiled when comparing groups are labeled as follows: Red and green shaded cells indicate p ≤ 0.05 (red specifies that the mean values are significantly higher for that comparison; green values significantly lower). The top 10 predictors were all lipids, which play a significant role in the brain as structural components of membranes and signaling molecules. Notably, there was an increase in prominent hexosylceramides, some phosphatidylcholines, lysoplasmalogen, some plasmalogens, and phosphatidylserine, whereas there was a decrease in other phosphatidylcholines, phosphatidylethanolamine, and some plasmalogen metabolites. Most of the observed changes were correlated with the known differences in white- and gray-enriched matter lipid populations due to the diverse functions of neuron cell biology. All analyses were performed in both gray-enriched matter and white-enriched matter to account for these differences.

### Machine learning to classify AD from normal with Gray and white-enriched matter metabolomics

Using random forest analysis, we found that the model predicted whether a sample came from AD brain tissue or normal brain tissue based on the metabolomics profile with 80.0% accuracy in the gray-enriched matter (Table 3B) and an 81.54% accuracy in the white-enriched matter (Table 3C). The top 9 predictors in gray-enriched matter mainly consisted of increases in phospholipid and creatine metabolism, decreases in amino acid metabolism, and the monohydroxy fatty acid 13-HODE + 9-HODE. The top 12 predictors in white-enriched matter mainly consisted of increases in phospholipid metabolism and decreases in amino acid metabolism, phosphatidylcholine, and some monohydroxy fatty acids. Glycerophosphocholine is formed in the breakdown of phosphatidylcholine and is increased in both the gray-enriched matter and white-enriched matter. N-acetylasparagine is a breakdown product of asparagine and is decreased in both the gray-enriched matter and white-enriched matter. The human body produces dimethylglycine when metabolizing choline into glycine, and it is decreased in the gray-enriched matter. N-acetyl-aspartyl-glutamate (NAAG) is a neuropeptide that is an agonist at mGluR3 receptors and an antagonist at NMDA receptors and is decreased in the gray-enriched matter. Pipecolic acid originates mainly from the catabolism of dietary lysine by intestinal bacteria rather than direct food intake, and it is decreased in both the gray-enriched matter and the white-enriched matter. Ureidopropionic acid is a urea compound and is an intermediate in the metabolism of uracil; it is decreased in the white-enriched matter.

Taken together, our findings indicate that glutamate and creatine metabolism is more important for predicting disease in the gray matter, while glycine, fatty acid, pyrimidine, tricarboxylic acid (TCA) cycle, and phosphatidylcholine metabolism are more important for predicting disease in the white matter.

### Gray and white-enriched matter metabolomics between early- and late-stage in *APOE* ε4

We next investigated the differences between late-stage and early-stage disease in the ε4 genotype (Table 4). We found that, compared with the early stage, late-stage *APOE* ε4 carriers had significantly reduced metabolite levels in the gray matter. Notable changes were found in pathways associated with mitochondrial function, glucose metabolism, and neurotransmitters, including the TCA cycle, oxidative phosphorylation, pentose metabolism, and acetyl-CoA and glutamate metabolism. We observed lower levels of serine and aspartate, whose declines correlate with the amount of Aβ plaques and neuronal pathology [14], as well as tyrosine and leucine, which are known to reduce atherosclerosis by improving the lipid profile and reducing systemic inflammation [15]. Further, metabolites related to mitigating oxidative stress, such as cysteine, arginine, gamma-glutamyl amino acid, pentose, were also lower in gray matter in the late stage. We also saw lipid decreases in seven lysophospholipid (LPL) species. LPL receptor ligands are known to bind to and activate their cognate receptors located in the cell membrane with a wide range of effects on the cell; these include the primary effects of inhibition of adenylyl cyclase and the release of calcium from the endoplasmic reticulum, as well as the secondary effects of preventing apoptosis and increasing cell proliferation [16].

In white matter, reductions were found in biochemicals related to glutamate, tyrosine, leucine, and methionine/cysteine metabolism. Interestingly, diacylglycerol was increased in the white matter in later disease similar to levels found in the ε3 genotype. Since diacylglycerol has been shown to reduce atherosclerosis in an *APOE*-deficient mouse model [17], the increase in diacylglycerol in the ε4 genotype could be a compensatory mechanism to combat rising levels of atherosclerosis.

The results show that with the disease progression, *APOE* ε4 carriers had alterations in metabolites associated with increased Aβ retention, reducing atherosclerosis, and the impaired TCA cycle and oxidative phosphorylation.

### Gray and white-enriched matter metabolomics between early- and late-stage in *APOE* ε3

We further investigated the differences between late-stage and early-stage in *APOE* ε3 genotype (Table 5). Individuals with ε3 variants had similar changes in the gray and white matters.

However, unlike ε4, which involves mitochondrial and glucose metabolism, notable key reductions in ε3 carriers were found in glycolysis, glutamate, tryptophan, and tyrosine metabolism. Glutamate is an excitatory neurotransmitter, which plays a critical role in learning and memory [18] and N-acetyl-aspartyl-glutamate (NAAG) has been shown to have precognitive effects by binding to metabotropic glutamate receptors [19]. Tryptophan metabolism is known to be altered in patients with AD [20], and tryptophan-derived metabolites can inhibit Aβ fibril formation in neurons and neuroblastoma cells [21]. Tryptophan is an essential amino acid and is the precursor of serotonin. Indole-3-propionic acid, a tryptophan-derived metabolite, can inhibit Aβ fibril formation in neurons and neuroblastoma cells [21]. Metabolites that play a role in tyrosine metabolism, including phenol sulfate, phenol glucuronide, and p-cresol glucuronide, are associated with inflammation [22].

We observed lower levels of dimethylglycine, which is linked to increased oxidative DNA damage associated with Aβ deposition [23], and homocarnosine, which is part of the histidine pathway and generally declines with age [24]. Neuronal histamine, phenylalanine, and tryptophan have a role in memory, reinforcement, and emotions [25–27]. We also found lower levels of fatty acids, which are often found as oxidized linoleic acid metabolites (OXLAMs) in the serum of AD patients [28] and phosphatidylcholine, which provides a reservoir of choline that can be used for acetylcholine synthesis. Notable key increases were found in myo-inositol metabolism, urea cycle, lysine, nucleotide sugar, inositol, and phospholipid. Myo-inositol, a neuroinflammatory marker, is negatively correlated with visuospatial working memory [29]. Lysine can act as a neurotransmitter modulating GABAergic transmission. The phospholipid is known to be raised in AD by disrupting neural cell membranes and causing cell death [30].

Taken together, our findings indicate that increases in metabolites linked to oxidative DNA damage, changes in inhibitory neurotransmitters, disruptions of neuronal membranes, and decreases in metabolites related to acetylcholine synthesis drive the differences between early- and late-stage of AD among *APOE* ε3 carriers.

### Gray and white-enriched matter metabolomics between *APOE* ε3 and *APOE* ε4 at early- and late-stage

We next compared the differences between *APOE* ε4 and 3 carriers at early-stage disease (Table 6). Notable changes were found in pathways associated with leucine, glycine, arginine, gamma-glutamyl amino acid, pentose, and secondary bile acid metabolism in *APOE* ε4 carriers compared to non-carriers. N,N,N-trimethyl-alanylproline betaine (TMAP), part of arginine metabolism, was lower in ε4 and is associated with poor kidney function [31]. We also found decreases in *glutathione*, commonly decreased with age [32], *eicosanoid*, and *sterol* - carriers of the *APOE* ε3/ε4 allele are known to exhibit altered neuronal sterol metabolism.

Unique differences in gray-enriched matter include an amino acid increase of histamine and 1-carboxyethylisoleucine (part of isoleucine metabolism), a peptide increase of gamma-glutamylisoleucine, a lipid increase of arachidonate (a long chain polyunsaturated fatty acid) and docosahexaenoyl ethanolamide (an endocannabinoid implicated in the pathology of neurodegenerative diseases).

Other differences were found only in the white-enriched matter. Unique differences include amino acid decreases in formiminoglutamate (part of histidine metabolism) and increases of 2-aminoadipate (part of lysine metabolism), lipid decreases in 1,2-dipalmitoyl-GPE (a phosphatidylethanolamine) and palmitoyl-docosahexaenoylglycerol (a diacylglycerol) and increases in 1,2-dioleoyl-GPG (a phosphatidylglycerol), 1-(1-enyl-oleoyl)-2-oleoyl-GPE (a lysoplasmalogen).

Taken together, our findings indicate that increased metabolites in *APOE* ε4 carriers related to poor kidney function and altered neuronal sterol metabolism drive the differences between the genotypes at an early stage.

Four detected metabolites increased between *APOE* ε4 and ε3 carriers at late stages of AD. At an alpha=0.01, a random chance would be expected to generate ~8 significant observations. Since our results do not surpass this threshold, our analyses did not identify metabolic differences between ε3 and ε4 carriers at late stages of AD.

## Discussion

We analyzed frontal cortical tissue from subjects across a spectrum of AD severity, with different genotypes (*APOE* 3/3 or *APOE* ε3/4) (summary of results is in Figure 2). We found distinct changes in the white versus gray-enriched matter of subjects as demonstrated through peptide alterations and lipid changes that have been associated with brain matter. There were increases in phospholipid metabolism and decreases in amino acid metabolism in AD brains compared with normal brains. Taken together, our findings indicated that glutamate and creatine metabolism were more important for predicting disease in the gray matter, and glycine, fatty acid, pyrimidine, TCA cycle, and phosphatidylcholine metabolism are more important for predicting disease in the white matter. As disease progressed in the *APOE* ε4 genotype, brains were characterized by decreases in metabolites responsible for reducing atherosclerosis and the TCA cycle and oxidative phosphorylation. With disease progression in the *APOE* ε3 genotype, brains were characterized by increases in metabolites related to oxidative DNA damage, changes in inhibitory neurotransmitters, disruptions of neuronal membranes, and decreases in metabolites related to acetylcholine synthesis. In early disease, the *APOE* 4 genotype was associated with increased metabolites related to poor kidney function and altered neuronal sterol metabolism, but there were few metabolic differences between *APOE* ε3 and ε4 genotypes in more severe AD.

The major differences between the metabolite composition of white-enriched matter and gray-enriched matter were characterized mainly by the metabolite components inherent to the myelin sheath present in the white-enriched matter. The myelin sheath is primarily comprised of lipids that insulate axons to speed action potentials, and it is not surprising that our data show that the top predictors for distinguishing gray-enriched matter from white-enriched matter were all lipids. Alpha-hydroxylated cerebrosides are the most abundant lipids in the myelin sheath [33], and the myelin sheath has lower phosphatidylcholine to phosphatidylethanolamine ratio compared with grey matter due to its unique composition of myelin, which is consistent with our data. It is crucial to separate white-enriched matter from gray-enriched matter in brain metabolomics analyses so that differences found between samples are not confounded by the inherent differences between tissue types (and sample-to-sample differences in ratios of white and gray matter).

Previous studies investigating metabolomics changes in AD reported changes in phosphatidylcholine and acylcarnitine metabolism [34], taurine transport, bile acid synthesis, and cholesterol metabolism [10,11,35], lipids, sphingolipids (notably GM_3_ gangliosides) and lipid classes previously associated with cardiometabolic disease (phosphatidylethanolamine and triglycerides) [36,37]. Results using Metabolon’s untargeted assay showed changes in not only phospholipid, phosphatidylcholine, and fatty acid metabolism, but also the TCA cycle, pyrimidine, and several amino acids including aspartate, lysine, glycine, glutamate, creatine, histidine. In addition to the lipid changes found by others, these critical changes in amino acid, energy, and nucleotide could give important clues about the underlying disease mechanism of AD.

Separating our analysis by Braak NFT stages and *APOE* genotype allowed us to better understand the nuances of metabolite changes unique to the disease stage and genotype. As the disease progresses in the *APOE* 4 carriers, metabolites associated with reducing atherosclerosis and the TCA cycle and oxidative phosphorylation appeared to correlate with (and perhaps to drive) the differences between early- and late-stage AD. Previous studies have reported that age-related vascular changes accompany or even precede the development of AD pathology [38], and a plant-based diet can reduce atherosclerosis [39]. Also, the impaired energy production is consistent with the findings from preclinical trials that *APOE* ε4 carriers experienced weakened glucose metabolism in the brain [40,41]. It suggests that intervention targeting impaired glucose metabolism may be critical for AD treatment, and early mediation in *APOE* ε4 carriers can be an essential path to avert the risk of AD.

Further, it has been observed that the TCA cycle can regulate the pathogenesis of neuroinflammation and neurodegeneration [42]. Interestingly, TCA cycle metabolite decreases are statistically more significant in the gray matter than the white matter in the ε4 genotype, but the machine learning analysis revealed TCA cycle metabolites to be more critical in predicting AD in the white matter. While TCA cycle decreases are more commonly seen in late-stage gray matter E4 in general, when TCA cycle decreases are seen in white matter, they are more likely to be predictive of AD, whereas other metabolites in the gray matter are more predictive of AD.

As AD progresses in the *APOE* ε3 genotype, metabolites linked to oxidative DNA damage, changes in inhibitory neurotransmitters, disruptions of neuronal membranes, and decreases in metabolites related to acetylcholine synthesis were correlated best with the differences between early- and late-stage AD. Oxidative stress may participate in AD development by promoting Aβ deposition, tau hyperphosphorylation, and the subsequent loss of synapses and neurons [43]. There is evidence that the Mediterranean diet is protective against oxidative DNA damage [44]. There is growing evidence supporting GABAergic remodeling in the AD brain, potentially beginning in early stages of disease pathogenesis [45]. Alterations of fatty acids at the level of lipid rafts and cerebral lipid peroxidation were found in the early stage of AD [46]. Cholinergic neurons located in the basal forebrain, including the neurons comprising the nucleus basalis of Meynert, are severely lost in AD [47].

Separating by *APOE* genotype was an essential part of our analysis as the *APOE* ε4 genotype is the most common genetic risk factor for developing AD. At the early stage of disease, *APOE* ε4 carriers differed from *APOE* ε3/3 in metabolites related to poor kidney function and altered neuronal sterol metabolism. Older patients on hemodialysis are at substantial risk of diagnosis with dementia and Alzheimer’s disease [48]. *APOE* ε4-expressing cultured astrocytes and neurons have reduced cholesterol and phospholipid secretion, decreased lipid-binding capacity, and increased intracellular degradation [49]. This is likely due to the changed domain interaction with cholesterol receptors and less stable conformation of the *APOE* ε4 genotype that changes its involvement in lipid metabolism and neurobiology, thereby impacting neuronal repair, remodeling, and degeneration [50]. There were far fewer metabolic differences between *APOE* ε3 and ε4 genotypes in later disease stages. Genotype differences may be pronounced during early disease, but the disease course causes both genotypes to exhibit a similar biochemical profile as the disease progresses and neurons are lost. These genotype differences should be further explored to determine whether precision interventions, like the consumption of inulin or the administration of rapamycin, could be implemented for ε4 carriers [51,52].

Taken together, our current findings and those of previous reports suggest that maintaining normal brain glucose metabolism is critical for cognitive resilience; therefore, therapeutic strategies for preventing or treating AD may need to shift focus from Aβ toward the preservation and restoration of normal brain metabolism. Recently, aducanumab, an Aβ directed antibody, was granted accelerated approval to verify its clinical benefit for use in early AD after decades of failed drugs targeted at Aβ; it is possible that metabolic interventions could be used across the disease course to provide clinical benefit to patients. Interventions with a metabolic therapeutic strategy have been reported that use intranasal insulin administration and a ketogenic diet. Regarding the potential benefits of a ketogenic diet, ketone bodies can function as an alternative fuel substrate in the brain when glucose is unavailable or when glucose metabolism is impaired due to insulin resistance [53–56]. One study showed that a ketogenic diet could modulate the deposition of Aβ and Tau in the CSF of MCI patients in conjunction with its modulation of the gut microbiome and the production of short-chain fatty acids [57]. This finding is consistent with an animal study showing that a ketogenic diet enhanced Aβ clearance across the blood-brain barrier and improved the composition of the gut microbiome [58].

The gut microbiome produces secondary bile acids. As mentioned above, alterations of bile acid production have been observed in AD patients due to gut microbiome imbalances, suggesting another mechanism by which AD patients may benefit from therapeutic strategies aiming to restore normal brain metabolism like the ketogenic diet [59,60]. Another animal study showed that by modulating the gut microbiome with a prebiotic diet, mice with the human *APOE* ε4 gene had enhanced systemic metabolism and reduced neuroinflammatory gene expression, another hallmark of AD pathology [51,61]. Collectively, modulating metabolic function and the gut microbiome may profoundly impact reducing the risk of AD.

This study is limited by the characterization of samples from only one area of the brain. Furthermore, our population consisted of participants with dementias from mixed pathologies. Future studies that evaluate the serum of subjects may be helpful to assess potential systemic metabolomic changes in subjects and if the differences observed here are limited to brain tissue. Similarly, analyses of cell-sorted tissue could give additional resolution to the results. Furthermore, minimizing drug usage in subjects (about 9% of biochemicals in the named dataset were pharmaceuticals that included AD therapies such as donepezil and memantine and the anti-diabetic medication metformin) may provide additional clarity to the above observations.

In conclusion, our study shows that metabolite differences are associated with disease stage, genotype, and cognitive decline in AD. Further investigation in AD metabolomics may elucidate new insights into disease mechanisms and therapeutics with precision medicine approaches.

## Supplementary Material

JCI-21-125_Supplementary Table 1

## Figures and Tables

**Table 1:** Participant Characteristics.

Number of Participants	158
Age	85.6 (84.4, 86.9)
APOE (% 4 carriers)	36.7%
Braak Stage 0–3	51.3%
Braak Stage 4–6	48.7%
Gender (% Female)	58.9%
Race (% White)	94.9%
Race (% Black)	5.1%
MMSE	22.2 (20.8, 23.6)
Postmortem Interval	3.7 (3.36, 4.02)
Consensus Diagnosis (% AD)	10.8%
Consensus Diagnosis (% Mixed AD)	42.7%
Consensus Diagnosis (% Other Dementia)	17.2%
Consensus Diagnosis (% Normal)	29.3%
TDP-43 (% Positive)	27.9%

**Table 2:** Number of samples belonging to APOE genotypes and Braak Stages.

	APOE ε3/ε3	APOE ε3/ε4	Total
**Braak Stage 0–3**	64	17	**81**
**Braak Stage 4–6**	36	41	**77**
**Total**	**100**	**58**	**158**

**Table 3:** Top ranked biochemicals in (A) predicting gray vs white matter all belong to the lipid superclass, (B) in predicting AD vs Normal brain tissue in Gray and (C) White Matter.

A) **Gray Matter vs White Matter**				
**Accuracy: 91.77%**				
**Rank Order**	**Super Pathway**	**Sub Pathway**	**Biochemical Name**	**Fold Change**
1	Lipid	Hexosylceramides (HCER)	glycosyl ceramide (d18:2/24:1, d18:1/24:2)*	**2.35**
2	Lipid	Phosphatidylcholine (PC)	1,2-dipalmitoyl-GPC (16:0/16:0)	**0.38**
3	Lipid	Phosphatidylcholine (PC)	1,2-dioleoyl-GPC (18:1/18:1)	**2.61**
4	Lipid	Lysoplasmalogen	1-(1-enyl-oleoyl)-2-oleoyl-GPE (P-18:1/18:1)*	**3.01**
5	Lipid	Plasmalogen	1-(1-enyl-palmitoyl)-2-oleoyl-GPE (P-16:0/18:1)*	**2.37**
6	Lipid	Hexosylceramides (HCER)	glycosyl ceramide (d18:2/25:1, d18:1/25:2)	**2.01**
7	Lipid	Phosphatidylethanolamine (PE)	1-palmitoyl-2-docosahexaenoyl-GPE (16:0/22:6)*	**0.41**
8	Lipid	Phosphatidylserine (PS)	1-stearoyl-2-oleoyl-GPS (18:0/18:1)	**2.11**
9	Lipid	Phosphatidylethanolamine (PE)	1-oleoyl-2-docosahexaenoyl-GPE (18:1/22:6)*	**0.54**
10	Lipid	Plasmalogen	1-(1-enyl-palmitoyl)-2-palmitoyl-GPC (P-16:0/16:0)*	**0.58**
B) **Gray Matter AD vs Normal**				
**Accuracy: 80.00%**				
**Rank Order**	**Super Pathway**	**Sub Pathway**	**Biochemical Name**	**Fold Change**
1	Lipid	Phospholipid Metabolism	glycerophosphorylcholine (GPC)	**1.51**
2	Amino Acid	Alanine and Aspartate Metabolism	N-acetylasparagine	**0.67**
3	Lipid	Fatty Acid, Monohydroxy	13-HODE + 9-HODE	**0.50**
4	Amino Acid	Lysine Metabolism	pipecolate	**0.47**
5	Lipid	Phospholipid Metabolism	glycerophosphoethanolamine	**1.26**
6	Amino Acid	Glycine, Serine and Threonine Metabolism	dimethylglycine	**0.58**
7	Amino Acid	Glutamate Metabolism	N-acetyl-aspartyl-glutamate (NAAG)	**0.75**
8	Amino Acid	Creatine Metabolism	Guanidinoacetate	**1.49**
9	Amino Acid	Histidine Metabolism	N-acetylhistidine	**0.57**
C) **White Matter AD vs Normal**				
**Accuracy: 81.54%**				
**Rank Order**	**Super Pathway**	**Sub Pathway**	**Biochemical Name**	**Fold Change**
1	Lipid	Fatty Acid, Monohydroxy	13-HODE + 9-HODE	**0.56**
2	Lipid	Phospholipid Metabolism	glycerophosphorylcholine (GPC)	**1.47**
3	Amino Acid	Lysine Metabolism	pipecolate	**0.48**
4	Amino Acid	Glycine, Serine and Threonine Metabolism	betaine	**0.74**
5	Amino Acid	Histidine Metabolism	N-acetylhistidine	**0.62**
6	Lipid	Fatty Acid, Monohydroxy	2-hydroxyheptanoate*	**0.81**
7	Lipid	Phospholipid Metabolism	glycerophosphoethanolamine	**1.25**
8	Nucleotide	Pyrimidine Metabolism, Uracil containing	3-ureidopropionate	**0.56**
9	Energy	TCA Cycle	2-methylcitrate/homocitrate	**0.80**
10	Lipid	Phosphatidylcholine (PC)	1,2-dilinoleoyl-GPC (18:2/18:2)	**0.74**
11	Amino Acid	Alanine and Aspartate Metabolism	N-acetylasparagine	**0.69**
12	Amino Acid	Glycine, Serine and Threonine Metabolism	dimethylglycine	**0.59**

Heat map of statistically significant biochemicals profiled when comparing groups are labeled as follows: Red and green shaded cells indicate *p* ≤ 0.05 (red specifies that the mean values are significantly higher for that comparison; green values significantly lower).

**Table 4:** Gray and white matter metabolomics between early and late stage in APOE4.

			E4 Late Stage vs Early Stage	
Super Pathway	Sub Pathway	Biochemical Name	Gray Matter	White Matter
Amino Acid	Glycine, Serine and Threonine Metabolism	N-acetylserine	**0.84**	**0.91**
	Alanine and Aspartate Metabolism	N-acetylasparagine	**0.74**	**0.80**
	Glutamate Metabolism	N-acetyl-aspartyl-glutamate (NAAG)	**0.72**	**0.70**
	Tyrosine Metabolism	4-hydroxyphenylpyruvate	0.81	**0.67**
	Leucine, Isoleucine and Valine Metabolism	methylsuccinate	**0.65**	**0.70**
		methylsuccinoylcarnitine	**0.73**	**0.72**
	Methionine, Cysteine, SAM and Taurine Metabolism	cystathionine	**0.80**	**0.72**
	Urea cycle; Arginine and Proline Metabolism	N-acetylarginine	**0.80**	0.93
Peptide	Gamma-glutamyl Amino Acid	gamma-glutamylisoleucine*	**0.63**	0.95
		gamma-glutamylmethionine	**0.68**	**0.73**
		gamma-glutamylthreonine	**0.81**	**0.96**
Carbohydrate	Pentose Metabolism	ribitol	**0.73**	**0.77**
	Fructose, Mannose and Galactose Metabolism	galactose 1-phosphate	**0.40**	1.01
Energy	TCA Cycle	fumarate	**0.80**	**0.86**
		malate	**0.81**	0.89
	Oxidative Phosphorylation	phosphate	**0.94**	**0.92**
Lipid	Fatty Acid Metabolism	acetyl CoA	**0.69**	**0.66**
	Phospholipid Metabolism	choline phosphate	**0.83**	**0.79**
	Lysophospholipid (LPL)	1-palmitoleoyl-GPC (16:1)*	**0.81**	0.94
		1-stearoyl-GPC (18:0)	**0.88**	0.92
		1-oleoyl-GPC (18:1)	**0.82**	0.91
		1-linoleoyl-GPC (18:2)	**0.79**	0.88
		1-arachidonoyl-GPC (20:4n6)*	**0.82**	1.01
		1-linoleoyl-GPE (18:2)*	**0.77**	0.94
		1-arachidonoyl-GPE (20:4n6)*	**0.80**	0.98
	Diacylglycerol	palmitoyl-docosahexaenoyl-glycerol (16:0/22:6) [1]*	1.26	**1.69**

Heat map of statistically significant biochemicals profiled when comparing groups are labeled as follows: Red and green shaded cells indicate *p* ≤ 0.05 (red specifies that the mean values are significantly higher for that comparison; green values significantly lower).

**Table 5:** Gray and white matter metabolomics between early and late stage in APOE3.

			E3 Late Stage vs Early Stage	
Super Pathway	Sub Pathway	Biochemical Name	Gray Matter	White Matter
Amino Acid	Glycine, Serine and Threonine Metabolism	N-acetylglycine	**1.75**	**1.62**
		dimethylglycine	**0.61**	**0.59**
		betaine	**0.71**	**0.69**
	Alanine and Aspartate Metabolism	N-acetylasparagine	**0.69**	**0.70**
	Glutamate Metabolism	N-acetyl-aspartyl-glutamate (NAAG)	**0.80**	0.88
		beta-citrylglutamate	**0.73**	**0.85**
	Histidine Metabolism	N-acetylhistidine	**0.62**	**0.67**
		N-acetyl-3-methylhistidine*	**1.48**	**1.48**
		homocarnosine	**0.67**	**0.74**
		1-methylhistamine	**0.65**	**0.67**
		1-methyl-4-imidazoleacetate	**0.63**	**0.82**
	Lysine Metabolism	2-aminoadipate	**1.21**	**1.24**
		pipecolate	**0.51**	**0.56**
		N-acetyl-2-aminoadipate	1.05	**1.15**
	Tyrosine Metabolism	3-methoxytyramine sulfate	**0.26**	**0.33**
	Tryptophan Metabolism	tryptophan betaine	**0.45**	**0.44**
		8-methoxykynurenate	**0.64**	**0.67**
		5-hydroxyindoleacetate	**0.62**	0.72
	Urea cycle; Arginine and Proline Metabolism	argininate*	**1.21**	**1.14**
	Polyamine Metabolism	N-acetylputrescine	**0.76**	**0.74**
		N1,N12-diacetylspermine	**0.21**	**0.32**
	Glutathione Metabolism	S-lactoylglutathione	**0.70**	**0.94**
Carbohydrate	Glycolysis, Gluconeogenesis, and Pyruvate Metabolism	fructose 1,6-diphosphate/glucose 1,6-diphosphate/myo-inositol diphosphates	**0.51**	0.92
		dihydroxyacetone phosphate (DHAP)	**0.57**	**0.79**
	Nucleotide Sugar	UDP-galactose	**1.51**	**1.47**
Lipid	Long Chain Polyunsaturated Fatty Acid (n3 and n6)	tetradecadienoate (14:2)*	**0.75**	**0.69**
	Fatty Acid, Dicarboxylate	octadecenedioate (C18:1-DC)	**0.55**	**0.59**
	Fatty Acid Metabolism (Acyl Carnitine, Short Chain)	acetylcarnitine (C2)	**0.86**	**0.70**
	Fatty Acid Metabolism (Acyl Carnitine, Polyunsaturated)	arachidonoylcarnitine (C20:4)	**0.65**	0.84
		docosahexaenoylcarnitine (C22:6)*	**0.52**	**0.53**
	Fatty Acid, Monohydroxy	2-hydroxyheptanoate*	**0.76**	**0.78**
		13-HODE + 9-HODE	**0.62**	**0.54**
	Inositol Metabolism	myo-inositol	**1.15**	**1.21**
	Phospholipid Metabolism	glycerophosphorylcholine (GPC)	**1.34**	**1.34**
		glycerophosphoethanolamine	**1.18**	**1.20**
	Phosphatidylcholine (PC)	1-palmitoyl-2-linoleoyl-GPC (16:0/18:2)	0.96	**0.90**
		1,2-dilinoleoyl-GPC (18:2/18:2)	**0.79**	**0.62**

Heat map of statistically significant biochemicals profiled when comparing groups are labeled as follows: Red and green shaded cells indicate *p* ≤ 0.05 (red specifies that the mean values are significantly higher for that comparison; green values significantly lower).

**Table 6:** Gray and white matter metabolomics between APOE3 and APOE4 at early and late stages.

**Early Stage**				
			E4/E3 Fold Change	
**Super Pathway**	**Sub Pathway**	**Biochemical Name**	**Gray Matter**	**White Matter**
Amino Acid	Glycine, Serine and Threonine Metabolism	dimethylglycine	**0.60**	**0.57**
	Histidine Metabolism	formiminoglutamate	0.03	**0.02**
		histamine	**1.28**	1.15
		1-methyl-4-imidazoleacetate	**0.64**	**0.53**
	Lysine Metabolism	2-aminoadipate	1.27	**1.28**
	Leucine, Isoleucine and Valine Metabolism	1-carboxyethylisoleucine	**1.40**	1.22
		methylsuccinate	**1.38**	**1.41**
		methylsuccinoylcarnitine	**1.36**	**1.42**
	Urea cycle; Arginine and Proline Metabolism	N,N,N-trimethyl-alanylproline betaine (TMaP)	**0.52**	**0.49**
		argininate*	**1.38**	**1.29**
	Glutathione Metabolism	4-hydroxy-nonenal-glutathione	**0.55**	**0.49**
Peptide	Gamma-glutamyl Amino Acid	gamma-glutamylisoleucine*	**1.81**	1.31
		gamma-glutamylmethionine	**1.31**	**1.24**
		gamma-glutamylthreonine	**1.54**	**1.47**
Carbohydrate	Pentose Metabolism	ribitol	**1.30**	**1.23**
Energy	Oxidative Phosphorylation	phosphate	**1.06**	**1.09**
Lipid	Long Chain Polyunsaturated Fatty Acid (n3 and n6)	arachidonate (20:4n6)	**1.19**	1.09
	Eicosanoid	15-HETE	**0.54**	**0.50**
	Endocannabinoid	docosahexaenoyl ethanolamide	**1.32**	0.99
	Phosphatidylethanolamine (PE)	1,2-dipalmitoyl-GPE (16:0/16:0)*	0.94	**0.73**
	Phosphatidylglycerol (PG)	1,2-dioleoyl-GPG (18:1/18:1)	0.55	**1.25**
	Lysophospholipid	1-oleoyl-GPC (18:1)	**1.11**	**1.09**
		1-palmitoyl-GPS (16:0)*	**1.36**	0.96
	Sterol	7-hydroxycholesterol (alpha or beta)	**0.42**	**0.42**
	Secondary Bile Acid Metabolism	glycodeoxycholate	**0.46**	**0.59**
**Late Stage**				
			**E4/E3 Fold Change**	
**Super Pathway**	**Sub Pathway**	**Biochemical Name**	**Gray Matter**	**White Matter**
Amino Acid	Tryptophan Metabolism	indolelactate	**2.58**	1.76
	Urea cycle; Arginine and Proline Metabolism	homoarginine	**1.66**	**1.53**
	Polyamine Metabolism	N1,N12-diacetylspermine	**6.95**	**10.26**
Lipid	Primary Bile Acid Metabolism	glycochenodeoxycholate	**2.67**	**3.68**

Heat map of statistically significant biochemicals profiled when comparing groups are labeled as follows: Red and green shaded cells indicate *p* ≤ 0.05 (red specifies that the mean values are significantly higher for that comparison; green values significantly lower).
