# Supplementary material for: Human Gray and White Matter Metabolomics to Differentiate APOE and Stage Dependent Changes in Alzheimer’s Disease
Source: J Cell Immunol. Author manuscript; Available in PMC 2022 Mar 8. (PMC8903196; doi:10.33696/immunology.3.123)
Supplement: JCI-21-125_Supplementary Table 1 [file NIHMS1768988-supplement-JCI-21-125_Supplementary_Table_1.docx]

| **Supplementary Table 1:** Metabolomics Differences in Gray and White Matter | | | | | | |
| --- | --- | --- | --- | --- | --- | --- |
|  |  |  | White Matter / Gray Matter | | | |
| **Super Pathway** | **Sub Pathway** | **Biochemical Name** | **BS 0-3 APOE 3/3** | **BS 0-3 APOE 3/4** | **BS 4-6 APOE 3/3** | **BS 4-6 APOE 3/4** |
| Amino Acid | Glycine, Serine and Threonine Metabolism | dimethylglycine | **0.82** | **0.78** | **0.80** | **0.85** |
|  |  | betaine | **0.78** | **0.81** | **0.76** | **0.80** |
|  |  | serine | **1.09** | 1.07 | **1.10** | **1.12** |
|  | Alanine and Aspartate Metabolism | N-acetylalanine | **0.94** | **0.90** | **0.90** | **0.93** |
|  |  | aspartate | **1.34** | **1.36** | **1.34** | **1.35** |
|  |  | N-acetylaspartate (NAA) | **0.79** | **0.71** | **0.74** | **0.80** |
|  |  | asparagine | **0.82** | **0.69** | **0.80** | **0.88** |
|  |  | N-acetylasparagine | **0.91** | **0.88** | **0.91** | 0.96 |
|  | Glutamate Metabolism | glutamate | **0.86** | **0.81** | **0.85** | **0.87** |
|  |  | N-acetylglutamate | **0.86** | **0.80** | **0.86** | **0.89** |
|  |  | N-acetylglutamine | **0.89** | **0.84** | **0.88** | **0.91** |
|  |  | pyroglutamine* | **1.14** | **1.17** | **1.14** | **1.15** |
|  |  | N-acetyl-aspartyl-glutamate (NAAG) | **1.39** | **1.44** | **1.52** | **1.41** |
|  |  | beta-citrylglutamate | **1.29** | **1.33** | **1.49** | **1.39** |
|  |  | gamma-aminobutyrate (GABA) | **0.65** | **0.58** | **0.61** | **0.67** |
|  |  | carboxyethyl-GABA | **1.35** | **1.39** | **1.39** | **1.35** |
|  | Histidine Metabolism | histidine | **0.96** | **0.92** | **0.94** | **0.88** |
|  |  | 3-methylhistidine | **1.29** | 1.25 | **1.08** | **1.24** |
|  |  | N-acetylhistidine | **0.82** | **0.88** | **0.89** | 0.99 |
|  |  | imidazole propionate | **0.84** | **0.82** | **0.66** | **0.91** |
|  |  | formiminoglutamate | **0.97** | 0.76 | **1.26** | 1.27 |
|  |  | carnosine | **2.48** | **2.60** | **2.44** | **2.17** |
|  |  | homocarnosine | **1.53** | **1.58** | **1.68** | **1.60** |
|  |  | histamine | **0.80** | **0.72** | **0.78** | **0.89** |
|  |  | 1-methyl-5-imidazoleacetate | **0.91** | **0.99** | **0.82** | **0.92** |
|  |  | 4-imidazoleacetate | **0.56** | **0.58** | **0.58** | **0.64** |
|  | Lysine Metabolism | lysine | **0.93** | **0.86** | **0.91** | **0.93** |
|  |  | N6-acetyllysine | **0.94** | **0.80** | **0.92** | 1.00 |
|  |  | N6-methyllysine | **1.18** | **1.16** | **1.19** | **1.19** |
|  |  | N6,N6-dimethyllysine | **1.19** | 1.12 | **1.24** | **1.24** |
|  |  | N6,N6,N6-trimethyllysine | **0.87** | **0.80** | **0.83** | **0.93** |
|  |  | hydroxy-N6,N6,N6-trimethyllysine* | **0.83** | **0.75** | **0.77** | **0.85** |
|  |  | 5-(galactosylhydroxy)-L-lysine | **1.21** | **1.25** | **1.15** | **1.19** |
|  |  | fructosyllysine | **1.04** | 1.06 | 1.04 | **1.09** |
|  |  | saccharopine | **1.18** | 1.01 | **1.08** | **1.24** |
|  |  | 2-aminoadipate | **1.40** | **1.42** | **1.44** | **1.33** |
|  |  | glutarylcarnitine (C5-DC) | **1.51** | **1.80** | **1.57** | **1.48** |
|  |  | pipecolate | **1.03** | 1.08 | **1.14** | **1.06** |
|  |  | N,N,N-trimethyl-5-aminovalerate | **1.17** | **1.26** | **1.15** | **1.20** |
|  |  | N-acetyl-2-aminoadipate | **1.41** | **1.47** | **1.55** | **1.44** |
|  | Phenylalanine Metabolism | phenylalanine | **0.95** | **0.87** | **0.93** | **0.96** |
|  |  | N-acetylphenylalanine | **0.93** | 0.98 | **0.91** | 0.99 |
|  |  | 1-carboxyethylphenylalanine | **0.91** | **0.80** | **0.91** | 0.97 |
|  | Tyrosine Metabolism | tyrosine | **0.94** | **0.85** | **0.92** | **0.93** |
|  |  | 4-hydroxyphenylpyruvate | 0.97 | 0.81 | **0.89** | **0.67** |
|  |  | phenol sulfate | **0.91** | **0.89** | **0.89** | 0.93 |
|  |  | vanillactate | **0.90** | 0.96 | 0.86 | 0.99 |
|  |  | 3-methoxytyrosine | **1.14** | 1.06 | 1.00 | **1.00** |
|  |  | O-methyltyrosine | **1.14** | 1.08 | **1.16** | **1.12** |
|  | Tryptophan Metabolism | C-glycosyltryptophan | **1.26** | **1.26** | **1.25** | **1.27** |
|  |  | kynurenine | **1.12** | 1.09 | **1.09** | **1.17** |
|  |  | 8-methoxykynurenate | **0.73** | **0.70** | **0.77** | **0.79** |
|  |  | 5-hydroxyindoleacetate | **1.12** | **1.27** | **1.30** | **1.34** |
|  | Leucine, Isoleucine and Valine Metabolism | leucine | **0.88** | **0.80** | **0.86** | **0.88** |
|  |  | N-acetylleucine | **0.82** | **0.70** | **0.77** | **0.82** |
|  |  | 1-carboxyethylleucine | **0.84** | **0.76** | **0.87** | **0.93** |
|  |  | 4-methyl-2-oxopentanoate | **0.84** | 0.73 | **0.70** | **0.55** |
|  |  | isovalerylcarnitine (C5) | 1.18 | **1.62** | **1.27** | **1.14** |
|  |  | beta-hydroxyisovalerate | **0.80** | **0.78** | **0.78** | **0.80** |
|  |  | 3-methylglutarylcarnitine (2) | **1.18** | **1.39** | **1.24** | **1.36** |
|  |  | isoleucine | **0.90** | **0.82** | **0.88** | **0.90** |
|  |  | 1-carboxyethylisoleucine | 0.95 | **0.83** | 0.96 | 1.02 |
|  |  | 2-methylbutyrylcarnitine (C5) | **1.24** | **1.23** | **1.18** | **1.13** |
|  |  | 3-hydroxy-2-ethylpropionate | **0.82** | **0.76** | **0.78** | **0.83** |
|  |  | methylsuccinate | **1.41** | **1.45** | **1.68** | **1.54** |
|  |  | methylsuccinoylcarnitine | **1.41** | **1.47** | **1.39** | **1.45** |
|  |  | valine | **0.90** | **0.76** | **0.87** | **0.87** |
|  |  | N-acetylvaline | **0.82** | **0.78** | **0.79** | **0.85** |
|  |  | 1-carboxyethylvaline | **0.91** | **0.84** | **0.95** | 0.99 |
|  |  | 3-methyl-2-oxobutyrate | **0.53** | **0.67** | **0.66** | **0.38** |
|  |  | alpha-hydroxyisovalerate | **0.91** | **0.92** | **0.84** | **0.92** |
|  |  | isobutyrylcarnitine (C4) | **1.73** | **1.94** | **1.41** | **1.37** |
|  |  | 3-hydroxyisobutyrate | **0.84** | 0.91 | **0.79** | **0.91** |
|  | Methionine, Cysteine, SAM and Taurine Metabolism | methionine | **0.92** | **0.81** | **0.92** | **0.93** |
|  |  | N-acetylmethionine | **0.74** | **0.66** | **0.75** | **0.79** |
|  |  | N-formylmethionine | **0.72** | **0.64** | **0.66** | **0.77** |
|  |  | N-acetylmethionine sulfoxide | **0.72** | **0.67** | **0.81** | **0.73** |
|  |  | S-adenosylmethionine (SAM) | **1.61** | **1.43** | **1.45** | **1.44** |
|  |  | S-adenosylhomocysteine (SAH) | **1.09** | 1.06 | **1.11** | **1.16** |
|  |  | 2,3-dihydroxy-5-methylthio-4-pentenoate (DMTPA)* | **1.23** | **1.29** | **1.23** | **1.29** |
|  |  | cystathionine | **1.93** | **2.19** | **2.19** | **1.97** |
|  |  | cysteine | **1.25** | **1.36** | 1.20 | **1.36** |
|  |  | lanthionine | **1.54** | **1.61** | **1.71** | **1.58** |
|  |  | taurine | **1.17** | **1.15** | **1.17** | **1.15** |
|  |  | taurocyamine | **2.34** | **2.44** | **2.50** | **1.96** |
|  |  | 3-sulfo-L-alanine | **0.67** | **0.49** | **0.73** | **0.53** |
|  | Urea cycle; Arginine and Proline Metabolism | arginine | **1.24** | **1.21** | **1.25** | **1.24** |
|  |  | argininosuccinate | 1.12 | 1.18 | **1.23** | **1.35** |
|  |  | urea | **0.85** | **0.88** | **0.84** | **0.87** |
|  |  | ornithine | **0.89** | **0.76** | **0.83** | **0.86** |
|  |  | 2-oxoarginine* | **0.92** | **0.75** | 0.94 | 0.97 |
|  |  | citrulline | **1.63** | **1.83** | **1.90** | **1.64** |
|  |  | homocitrulline | **0.91** | 0.85 | 0.79 | **0.77** |
|  |  | proline | **0.90** | **0.87** | **0.94** | **0.92** |
|  |  | N-acetylarginine | **0.73** | **0.66** | **0.71** | **0.77** |
|  |  | N-delta-acetylornithine | **0.90** | 0.96 | **0.89** | 0.96 |
|  |  | trans-4-hydroxyproline | **1.22** | **1.23** | **1.18** | **1.18** |
|  |  | N,N,N-trimethyl-alanylproline betaine (TMAP) | **0.79** | **0.74** | **0.70** | **0.82** |
|  |  | argininate* | **1.33** | **1.24** | **1.25** | **1.22** |
|  | Creatine Metabolism | guanidinoacetate | **0.91** | **0.69** | **0.67** | **0.79** |
|  |  | creatinine | **1.06** | **1.06** | **1.06** | **1.08** |
|  |  | creatine phosphate | **0.91** | 1.08 | **0.77** | **0.53** |
|  | Polyamine Metabolism | putrescine | **0.76** | **0.61** | **0.65** | **0.80** |
|  |  | N-acetyl-isoputreanine | **0.69** | 0.83 | **0.86** | **0.86** |
|  |  | spermidine | **1.33** | **1.16** | **1.44** | **1.33** |
|  |  | diacetylspermidine* | **1.29** | **1.27** | **1.39** | **1.45** |
|  |  | acisoga | **0.82** | **0.82** | **0.76** | **0.86** |
|  |  | spermine | **0.34** | **0.20** | **0.30** | **0.39** |
|  |  | 5-methylthioadenosine (MTA) | **1.43** | **1.27** | **1.34** | **1.33** |
|  |  | 4-acetamidobutanoate | **1.08** | 1.01 | **1.15** | **1.16** |
|  | Guanidino and Acetamido Metabolism | 1-methylguanidine | **0.85** | **0.73** | **0.86** | **0.85** |
|  |  | 4-guanidinobutanoate | **1.20** | **1.16** | **1.21** | **1.22** |
|  | Glutathione Metabolism | glutathione, reduced (GSH) | **1.34** | **1.47** | **1.33** | **1.52** |
|  |  | glutathione, oxidized (GSSG) | **0.95** | **0.89** | **0.92** | **0.87** |
|  |  | S-methylglutathione | **1.29** | **1.23** | **1.34** | **1.34** |
|  |  | cysteinylglycine | **1.22** | 1.06 | 1.01 | **1.13** |
|  |  | 5-oxoproline | **1.09** | **1.14** | **1.13** | **1.22** |
|  |  | ophthalmate | **0.61** | **0.55** | **0.56** | **0.66** |
|  |  | S-(1,2-dicarboxyethyl)glutathione | **1.20** | **1.30** | **1.25** | **1.30** |
|  |  | 4-hydroxy-nonenal-glutathione | **0.91** | 0.81 | **0.84** | 0.93 |
|  |  | 3'-dephospho-CoA-glutathione* | **1.52** | **1.55** | **1.64** | **1.31** |
|  |  | CoA-glutathione* | **1.66** | **1.76** | **1.83** | **1.61** |
| Peptide | Gamma-glutamyl Amino Acid | gamma-glutamylcysteine | **3.67** | **4.35** | **4.03** | **3.65** |
|  |  | gamma-glutamylglutamate | **0.87** | 1.02 | **0.89** | **0.89** |
|  |  | gamma-glutamylglutamine | **1.19** | **1.38** | **1.25** | **1.31** |
|  |  | gamma-glutamylisoleucine* | **0.72** | **0.52** | **0.73** | **0.78** |
|  |  | gamma-glutamylleucine | **0.75** | **0.64** | **0.78** | **0.79** |
|  |  | gamma-glutamyl-epsilon-lysine | **1.93** | **1.92** | **2.28** | **2.03** |
|  |  | gamma-glutamylmethionine | **0.87** | **0.82** | **0.93** | **0.89** |
|  |  | gamma-glutamylthreonine | **0.78** | **0.74** | **0.82** | **0.88** |
|  |  | gamma-glutamylvaline | **0.67** | **0.60** | **0.66** | **0.69** |
|  | Dipeptide | glycylleucine | **0.54** | **0.33** | **0.45** | **0.53** |
|  |  | glycylvaline | **0.74** | **0.52** | **0.64** | **0.72** |
|  |  | isoleucylglycine | **0.73** | **0.38** | **0.51** | **0.65** |
|  |  | leucylglycine | **0.77** | **0.50** | **0.57** | **0.75** |
|  |  | phenylalanylalanine | **0.46** | **0.20** | **0.35** | **0.47** |
|  |  | phenylalanylglycine | 1.00 | **0.69** | **0.78** | **0.80** |
|  |  | threonylphenylalanine | **0.79** | **0.42** | **0.53** | **0.69** |
|  |  | tyrosylglycine | **1.23** | 1.07 | **1.19** | **1.11** |
|  |  | valylglutamine | **0.96** | **0.19** | **0.53** | **0.55** |
|  |  | valylglycine | **0.67** | **0.22** | **0.42** | **0.50** |
|  |  | valylleucine | **1.24** | **0.33** | **0.62** | **0.67** |
|  |  | leucylglutamine* | **0.60** | **0.34** | **0.50** | **0.57** |
| Carbohydrate | Glycolysis, Gluconeogenesis, and Pyruvate Metabolism | 1,5-anhydroglucitol (1,5-AG) | **0.85** | **0.88** | **0.85** | **0.88** |
|  |  | glucose | **0.60** | 0.52 | **0.44** | **0.72** |
|  |  | glucose 6-phosphate | **0.82** | **0.72** | **0.70** | **0.63** |
|  |  | fructose 1,6-diphosphate/glucose 1,6-diphosphate/myo-inositol diphosphates | **2.34** | **2.25** | **4.26** | **3.00** |
|  |  | dihydroxyacetone phosphate (DHAP) | **1.22** | **1.22** | **1.68** | **1.38** |
|  |  | 3-phosphoglycerate | **0.31** | **0.26** | **0.33** | **0.34** |
|  |  | phosphoenolpyruvate (PEP) | **0.26** | **0.21** | **0.26** | **0.29** |
|  |  | pyruvate | **0.66** | **0.51** | **0.64** | **0.68** |
|  |  | lactate | **0.92** | **0.84** | **0.91** | 0.95 |
|  |  | glycerate | **0.72** | **0.71** | **0.72** | **0.77** |
|  | Pentose Phosphate Pathway | ribose 1-phosphate | **1.23** | 1.16 | **1.28** | **1.17** |
|  |  | sedoheptulose-7-phosphate | **0.51** | **0.44** | **0.49** | **0.54** |
|  | Pentose Metabolism | ribose | **0.82** | **0.74** | **0.81** | **0.84** |
|  |  | ribitol | **0.89** | **0.85** | **0.91** | **0.90** |
|  |  | ribonate | 1.05 | 1.06 | 1.04 | **1.18** |
|  |  | ribulose/xylulose | **0.63** | **0.52** | **0.55** | **0.63** |
|  |  | arabinose | 0.97 | **0.87** | **0.92** | 0.96 |
|  |  | arabitol/xylitol | **0.89** | **0.87** | **0.88** | **0.93** |
|  |  | sedoheptulose | **0.57** | **0.54** | **0.62** | **0.69** |
|  |  | ribulonate/xylulonate/lyxonate* | **0.81** | **0.77** | **0.82** | **0.92** |
|  | Fructose, Mannose and Galactose Metabolism | fructose | **0.62** | **0.53** | **0.53** | **0.70** |
|  |  | mannitol/sorbitol | **0.83** | **0.82** | **0.83** | **0.93** |
|  |  | mannose | **0.60** | **0.46** | **0.55** | **0.70** |
|  |  | galactose 1-phosphate | **1.39** | 0.80 | 0.97 | **2.02** |
|  | Nucleotide Sugar | UDP-glucose | **1.31** | 1.08 | **1.11** | **1.10** |
|  |  | UDP-galactose | **1.65** | **1.35** | **1.61** | **1.44** |
|  |  | UDP-N-acetylglucosamine/galactosamine | **1.31** | **1.42** | **1.38** | **1.35** |
|  | Aminosugar Metabolism | N-acetylneuraminate | **0.66** | **0.57** | **0.63** | **0.70** |
|  |  | N-acetylglucosaminylasparagine | **0.88** | **0.83** | **0.84** | **0.85** |
|  |  | N-acetylglucosamine/N-acetylgalactosamine | **0.91** | **0.86** | **0.88** | **0.94** |
|  | Advanced Glycation End-product | N6-carboxymethyllysine | **1.48** | **1.49** | **1.56** | **1.49** |
| Energy | TCA Cycle | aconitate [cis or trans] | **0.91** | **0.87** | **0.82** | **0.91** |
|  |  | alpha-ketoglutarate | **0.77** | **0.76** | **0.73** | **0.77** |
|  |  | succinylcarnitine (C4-DC) | **1.28** | **1.34** | **1.42** | **1.36** |
|  |  | succinate | **2.18** | **2.63** | **1.95** | **1.77** |
|  |  | fumarate | **0.75** | **0.74** | **0.79** | **0.79** |
|  |  | malate | **0.83** | **0.79** | **0.88** | **0.88** |
|  | Oxidative Phosphorylation | phosphate | **1.08** | **1.11** | **1.10** | **1.08** |
| Lipid | Fatty Acid Metabolism | acetyl CoA | **1.38** | **1.47** | **1.25** | **1.40** |
|  | Medium Chain Fatty Acid | caproate (6:0) | **0.89** | 0.95 | **0.85** | **0.78** |
|  |  | caprate (10:0) | **0.81** | **0.86** | **0.92** | **0.91** |
|  | Long Chain Saturated Fatty Acid | myristate (14:0) | **0.91** | 1.01 | **0.88** | **0.90** |
|  |  | pentadecanoate (15:0) | **0.91** | 0.96 | 0.90 | **0.89** |
|  |  | palmitate (16:0) | **0.86** | **0.91** | **0.83** | **0.86** |
|  |  | stearate (18:0) | **0.83** | **0.84** | **0.79** | **0.83** |
|  | Long Chain Monounsaturated Fatty Acid | myristoleate (14:1n5) | **0.89** | 1.08 | **0.86** | **0.90** |
|  |  | 10-heptadecenoate (17:1n7) | **1.76** | **2.45** | **1.97** | **1.75** |
|  |  | oleate/vaccenate (18:1) | **1.19** | 1.47 | **1.24** | **1.22** |
|  |  | 10-nonadecenoate (19:1n9) | **1.85** | **2.52** | **2.00** | **1.84** |
|  |  | eicosenoate (20:1) | **1.61** | **2.17** | **1.74** | **1.59** |
|  |  | erucate (22:1n9) | **1.90** | **2.65** | **2.08** | **1.76** |
|  | Long Chain Polyunsaturated Fatty Acid (n3 and n6) | tetradecadienoate (14:2)* | **0.86** | 0.98 | **0.79** | **0.89** |
|  |  | eicosapentaenoate (EPA; 20:5n3) | **0.61** | **0.58** | **0.61** | **0.62** |
|  |  | docosapentaenoate (n3 DPA; 22:5n3) | **0.72** | **0.80** | **0.64** | **0.69** |
|  |  | docosahexaenoate (DHA; 22:6n3) | **0.55** | **0.51** | **0.46** | **0.53** |
|  |  | nisinate (24:6n3) | **0.67** | **0.78** | **0.58** | **0.63** |
|  |  | hexadecadienoate (16:2n6) | **0.60** | **0.70** | **0.51** | **0.56** |
|  |  | linolenate [alpha or gamma; (18:3n3 or 6)] | **0.75** | **0.95** | **0.71** | **0.75** |
|  |  | dihomo-linoleate (20:2n6) | **1.46** | **1.98** | **1.47** | **1.41** |
|  |  | dihomo-linolenate (20:3n3 or n6) | **1.30** | 1.82 | **1.24** | 1.20 |
|  |  | arachidonate (20:4n6) | **0.86** | **0.79** | **0.86** | **0.92** |
|  |  | docosatrienoate (22:3n6)* | **2.86** | **3.79** | **3.32** | **2.69** |
|  |  | docosapentaenoate (n6 DPA; 22:5n6) | **0.84** | 1.09 | **0.71** | **0.88** |
|  |  | docosadienoate (22:2n6) | **2.12** | **3.02** | **2.32** | **1.97** |
|  |  | mead acid (20:3n9) | **2.46** | **3.41** | **2.70** | **2.54** |
|  | Fatty Acid, Dicarboxylate | 2-hydroxyglutarate | **1.08** | 1.06 | **1.18** | **1.20** |
|  |  | 2-hydroxyadipate | **1.36** | **1.38** | **1.42** | **1.35** |
|  |  | maleate | **0.89** | 0.90 | 0.96 | **0.91** |
|  | Fatty Acid Metabolism (also BCAA Metabolism) | butyrylcarnitine (C4) | **0.87** | **0.81** | **0.80** | **0.88** |
|  |  | propionylcarnitine (C3) | **0.79** | **0.78** | **0.80** | **0.84** |
|  |  | methylmalonate (MMA) | **0.76** | **0.66** | **0.78** | **0.88** |
|  | Fatty Acid Metabolism (Acyl Carnitine, Short Chain) | acetylcarnitine (C2) | **0.80** | **0.70** | **0.66** | **0.78** |
|  | Fatty Acid Metabolism (Acyl Carnitine, Medium Chain) | hexanoylcarnitine (C6) | **0.82** | **0.76** | **0.74** | 0.90 |
|  |  | octanoylcarnitine (C8) | **0.99** | 1.08 | 1.07 | 1.31 |
|  |  | decanoylcarnitine (C10) | **2.42** | **3.32** | **2.83** | **2.86** |
|  |  | laurylcarnitine (C12) | **2.53** | **3.49** | **3.12** | **2.84** |
|  | Fatty Acid Metabolism (Acyl Carnitine, Long Chain Saturated) | myristoylcarnitine (C14) | **2.70** | **3.56** | **3.64** | **3.19** |
|  |  | pentadecanoylcarnitine (C15)* | **2.32** | **3.14** | **3.11** | **2.78** |
|  |  | palmitoylcarnitine (C16) | **1.54** | **2.09** | **1.93** | **1.94** |
|  |  | margaroylcarnitine (C17)* | **1.22** | **1.33** | **1.38** | **1.34** |
|  |  | stearoylcarnitine (C18) | **0.92** | 0.96 | 0.96 | 0.98 |
|  |  | arachidoylcarnitine (C20)* | **0.79** | **0.79** | **0.79** | **0.81** |
|  |  | behenoylcarnitine (C22)* | **0.66** | **0.69** | **0.70** | **0.72** |
|  |  | lignoceroylcarnitine (C24)* | **0.55** | **0.63** | **0.52** | **0.54** |
|  | Fatty Acid Metabolism (Acyl Carnitine, Monounsaturated) | 5-dodecenoylcarnitine (C12:1) | **2.37** | **4.04** | **2.09** | **2.75** |
|  |  | myristoleoylcarnitine (C14:1)* | **2.11** | **3.44** | **2.93** | **2.71** |
|  |  | palmitoleoylcarnitine (C16:1)* | **1.99** | **2.88** | **2.66** | **2.37** |
|  |  | oleoylcarnitine (C18:1) | **1.57** | **2.12** | **1.95** | **1.96** |
|  |  | eicosenoylcarnitine (C20:1)* | **0.92** | 1.15 | 0.95 | 1.01 |
|  |  | erucoylcarnitine (C22:1)* | **0.87** | 1.12 | 0.92 | 0.97 |
|  |  | nervonoylcarnitine (C24:1)* | **0.88** | 1.07 | 0.95 | 1.01 |
|  |  | ximenoylcarnitine (C26:1)* | **1.66** | **1.93** | **1.65** | **1.76** |
|  | Fatty Acid Metabolism (Acyl Carnitine, Polyunsaturated) | linoleoylcarnitine (C18:2)* | **1.31** | **1.52** | **1.37** | **1.50** |
|  |  | linolenoylcarnitine (C18:3)* | **1.64** | **2.39** | **1.56** | **1.98** |
|  |  | dihomo-linoleoylcarnitine (C20:2)* | **0.84** | **0.92** | **0.79** | **0.88** |
|  |  | arachidonoylcarnitine (C20:4) | **2.00** | **2.86** | **2.58** | **2.63** |
|  |  | dihomo-linolenoylcarnitine (C20:3n3 or 6)* | **1.55** | **1.97** | **1.68** | **1.81** |
|  |  | docosadienoylcarnitine (C22:2)* | **0.77** | **0.85** | **0.70** | **0.75** |
|  |  | docosatrienoylcarnitine (C22:3)* | **0.66** | **0.74** | **0.61** | **0.70** |
|  |  | tetracosadienoylcarnitine (C24:2)* | **0.67** | **0.73** | **0.67** | **0.72** |
|  | Fatty Acid Metabolism (Acyl Carnitine, Hydroxy) | (R)-3-hydroxybutyrylcarnitine | **1.17** | **1.39** | **1.23** | **1.25** |
|  |  | cis-3,4-methyleneheptanoylcarnitine | **0.77** | 0.94 | **0.66** | **0.78** |
|  |  | 3-hydroxydecanoylcarnitine | **1.70** | **2.36** | **1.62** | **1.82** |
|  | Neurotransmitter | acetylcholine | **0.45** | **0.41** | **0.41** | **0.35** |
|  | Fatty Acid, Monohydroxy | 2-hydroxyheptanoate* | **0.89** | 0.96 | 0.91 | **0.83** |
|  |  | 2-hydroxystearate | **1.26** | **1.74** | **1.46** | **1.38** |
|  |  | 2-hydroxybehenate | **2.26** | **2.75** | **2.68** | **1.92** |
|  |  | 2-hydroxynervonate* | **3.09** | **4.12** | **4.27** | **2.64** |
|  |  | 3-hydroxyhexanoate | **0.88** | **0.81** | **0.85** | **0.90** |
|  |  | 3-hydroxylaurate | **1.12** | **1.17** | **1.12** | **1.17** |
|  |  | 3-hydroxymyristate | **0.94** | 1.03 | **0.93** | 0.98 |
|  |  | 13-HODE + 9-HODE | **0.79** | 0.79 | **0.69** | **0.67** |
|  | Fatty Acid, Dihydroxy | 2S,3R-dihydroxybutyrate | **0.82** | **0.85** | **0.81** | **0.86** |
|  |  | 2,4-dihydroxybutyrate | **0.84** | **0.88** | **0.88** | **0.92** |
|  | Eicosanoid | 15-HETE | **0.69** | **0.64** | **0.65** | **0.74** |
|  | Endocannabinoid | oleoyl ethanolamide | **1.67** | **2.12** | **1.93** | **1.93** |
|  |  | palmitoyl ethanolamide | **1.89** | **2.27** | **2.15** | **1.99** |
|  |  | stearoyl ethanolamide | **0.90** | 0.96 | **0.89** | 0.99 |
|  |  | docosahexaenoyl ethanolamide | **0.70** | **0.53** | **0.66** | **0.73** |
|  |  | arachidonoyl ethanolamide | **0.60** | **0.52** | **0.56** | **0.63** |
|  |  | N-oleoyltaurine | **1.63** | **2.03** | **1.87** | **1.64** |
|  |  | N-stearoyltaurine | **1.39** | **1.68** | **1.47** | **1.32** |
|  |  | N-palmitoyltaurine | **1.81** | **2.38** | **2.28** | **1.79** |
|  |  | linoleoyl ethanolamide | **0.81** | **0.97** | **0.70** | **0.80** |
|  |  | palmitoleoyl ethanolamide* | **1.74** | **2.06** | **1.95** | **1.81** |
|  |  | ximenoyl ethanolamide (26:1)* | **3.82** | **5.42** | **5.58** | **3.65** |
|  |  | N-oleoylserine | **2.13** | **2.77** | **2.34** | **2.07** |
|  |  | N-stearoylserine* | **1.42** | **1.71** | **1.48** | **1.44** |
|  |  | N-palmitoylserine | **1.86** | **2.35** | **2.08** | **1.89** |
|  | Inositol Metabolism | myo-inositol | **1.17** | **1.16** | **1.23** | **1.19** |
|  |  | chiro-inositol | **1.40** | 1.36 | **1.52** | **1.54** |
|  | Phospholipid Metabolism | choline | **1.06** | **1.13** | **1.09** | **1.12** |
|  |  | choline phosphate | **1.07** | **1.17** | **1.19** | **1.12** |
|  |  | cytidine 5'-diphosphocholine | **1.28** | **1.28** | **1.28** | **1.27** |
|  |  | phosphoethanolamine | **0.69** | **0.66** | **0.65** | **0.72** |
|  |  | cytidine-5'-diphosphoethanolamine | **1.08** | **1.09** | **1.11** | **1.10** |
|  |  | glycerophosphoethanolamine | **1.11** | **1.10** | **1.13** | **1.12** |
|  |  | glycerophosphoserine* | **0.94** | **0.94** | 0.95 | 0.98 |
|  |  | glycerophosphoinositol* | **0.90** | 0.95 | 0.97 | 0.99 |
|  |  | trimethylamine N-oxide | **0.91** | **0.89** | **0.81** | **0.90** |
|  | Phosphatidylcholine (PC) | 1-myristoyl-2-palmitoyl-GPC (14:0/16:0) | **0.70** | **0.62** | **0.66** | **0.72** |
|  |  | 1-myristoyl-2-arachidonoyl-GPC (14:0/20:4)* | **0.77** | **0.68** | **0.72** | **0.78** |
|  |  | 1,2-dipalmitoyl-GPC (16:0/16:0) | **0.41** | **0.32** | **0.36** | **0.41** |
|  |  | 1-palmitoyl-2-stearoyl-GPC (16:0/18:0) | **0.29** | **0.22** | **0.23** | **0.29** |
|  |  | 1-palmitoyl-2-oleoyl-GPC (16:0/18:1) | **0.98** | **0.96** | **0.98** | 1.00 |
|  |  | 1-palmitoyl-2-linoleoyl-GPC (16:0/18:2) | **0.89** | **0.84** | **0.83** | **0.87** |
|  |  | 1-palmitoyl-2-gamma-linolenoyl-GPC (16:0/18:3n6)* | **0.63** | **0.56** | **0.59** | **0.66** |
|  |  | 1-palmitoyl-2-arachidonoyl-GPC (16:0/20:4n6) | **0.67** | **0.60** | **0.62** | **0.68** |
|  |  | 1-palmitoyl-2-docosahexaenoyl-GPC (16:0/22:6) | **0.54** | **0.46** | **0.49** | **0.54** |
|  |  | 1-stearoyl-2-oleoyl-GPC (18:0/18:1) | **1.15** | **1.14** | **1.18** | **1.17** |
|  |  | 1-stearoyl-2-arachidonoyl-GPC (18:0/20:4) | **0.65** | **0.58** | **0.60** | **0.66** |
|  |  | 1-stearoyl-2-docosahexaenoyl-GPC (18:0/22:6) | **0.51** | **0.44** | **0.47** | **0.52** |
|  |  | 1,2-dioleoyl-GPC (18:1/18:1) | **2.34** | **2.80** | **2.89** | **2.40** |
|  |  | 1-oleoyl-2-docosahexaenoyl-GPC (18:1/22:6)* | **0.59** | **0.52** | **0.55** | **0.60** |
|  |  | 1-linoleoyl-2-arachidonoyl-GPC (18:2/20:4n6)* | **0.74** | **0.62** | **0.65** | **0.72** |
|  | Phosphatidylethanolamine (PE) | 1,2-dipalmitoyl-GPE (16:0/16:0)* | **0.17** | **0.13** | **0.14** | **0.18** |
|  |  | 1-palmitoyl-2-oleoyl-GPE (16:0/18:1) | **0.69** | **0.66** | **0.67** | **0.74** |
|  |  | 1-palmitoyl-2-arachidonoyl-GPE (16:0/20:4)* | **0.52** | **0.46** | **0.48** | **0.55** |
|  |  | 1-palmitoyl-2-docosahexaenoyl-GPE (16:0/22:6)* | **0.44** | **0.37** | **0.39** | **0.45** |
|  |  | 1-stearoyl-2-oleoyl-GPE (18:0/18:1) | **0.66** | **0.61** | **0.65** | **0.72** |
|  |  | 1-stearoyl-2-arachidonoyl-GPE (18:0/20:4) | **0.48** | **0.40** | **0.42** | **0.49** |
|  |  | 1-stearoyl-2-docosahexaenoyl-GPE (18:0/22:6)* | **0.42** | **0.33** | **0.35** | **0.42** |
|  |  | 1,2-dioleoyl-GPE (18:1/18:1) | **1.34** | **1.48** | **1.48** | **1.39** |
|  |  | 1-oleoyl-2-linoleoyl-GPE (18:1/18:2)* | **0.84** | **0.80** | **0.79** | **0.84** |
|  |  | 1-oleoyl-2-arachidonoyl-GPE (18:1/20:4)* | **0.86** | **0.84** | **0.86** | **0.89** |
|  |  | 1-oleoyl-2-docosahexaenoyl-GPE (18:1/22:6)* | **0.56** | **0.49** | **0.53** | **0.57** |
|  |  | 1-linoleoyl-2-arachidonoyl-GPE (18:2/20:4)* | **0.78** | **0.76** | **0.75** | **0.79** |
|  | Phosphatidylserine (PS) | 1-palmitoyl-2-oleoyl-GPS (16:0/18:1) | **0.69** | **0.61** | **0.62** | **0.66** |
|  |  | 1-stearoyl-2-oleoyl-GPS (18:0/18:1) | **2.03** | **2.15** | **2.27** | **1.97** |
|  |  | 1-stearoyl-2-arachidonoyl-GPS (18:0/20:4) | **1.15** | 1.08 | **1.12** | **1.10** |
|  |  | 1,2-dioleoyl-GPS (18:1/18:1) | **2.00** | **2.34** | **2.29** | **2.02** |
|  | Phosphatidylglycerol (PG) | 1,2-dipalmitoyl-GPG (16:0/16:0) | **0.24** | **0.15** | **0.15** | **0.24** |
|  |  | 1-palmitoyl-2-oleoyl-GPG (16:0/18:1) | **0.55** | **0.47** | **0.49** | **0.54** |
|  |  | 1,2-dioleoyl-GPG (18:1/18:1) | **3.67** | **8.25** | **4.47** | **4.58** |
|  | Phosphatidylinositol (PI) | 1-palmitoyl-2-oleoyl-GPI (16:0/18:1)* | **1.47** | **1.55** | **1.81** | **1.66** |
|  |  | 1-palmitoyl-2-arachidonoyl-GPI (16:0/20:4)* | **0.52** | **0.46** | **0.49** | **0.56** |
|  |  | 1-stearoyl-2-arachidonoyl-GPI (18:0/20:4) | **0.67** | **0.62** | **0.60** | **0.66** |
|  |  | 1-oleoyl-2-arachidonoyl-GPI (18:1/20:4)* | **0.90** | **0.83** | **0.85** | **0.86** |
|  | Lysophospholipid | 1-arachidonoyl-GPA (20:4) | **0.81** | **0.81** | 0.92 | 0.99 |
|  |  | 1-palmitoyl-GPC (16:0) | **0.86** | **0.81** | **0.85** | **0.90** |
|  |  | 2-palmitoyl-GPC (16:0)* | **0.79** | **0.77** | **0.74** | **0.76** |
|  |  | 1-palmitoleoyl-GPC (16:1)* | **0.79** | **0.72** | **0.79** | **0.83** |
|  |  | 2-palmitoleoyl-GPC (16:1)* | **0.52** | 0.40 | **0.50** | **0.47** |
|  |  | 1-oleoyl-GPC (18:1) | **0.93** | **0.91** | 0.99 | 1.01 |
|  |  | 1-linoleoyl-GPC (18:2) | **0.77** | **0.69** | **0.74** | **0.77** |
|  |  | 1-arachidonoyl-GPC (20:4n6)* | **0.60** | **0.52** | **0.58** | **0.64** |
|  |  | 1-lignoceroyl-GPC (24:0) | **2.88** | **4.24** | **3.50** | **3.05** |
|  |  | 1-cerotoyl-GPC (26:0)* | **4.28** | **5.72** | **5.68** | **4.38** |
|  |  | 1-palmitoyl-GPE (16:0) | **0.60** | **0.53** | **0.56** | **0.62** |
|  |  | 1-stearoyl-GPE (18:0) | **0.50** | **0.40** | **0.44** | **0.50** |
|  |  | 2-stearoyl-GPE (18:0)* | **0.54** | **0.44** | **0.47** | **0.52** |
|  |  | 1-oleoyl-GPE (18:1) | **1.28** | **1.45** | **1.51** | **1.48** |
|  |  | 1-linoleoyl-GPE (18:2)* | **0.64** | **0.62** | **0.66** | **0.75** |
|  |  | 1-arachidonoyl-GPE (20:4n6)* | **0.60** | **0.55** | **0.61** | **0.68** |
|  |  | 1-palmitoyl-GPS (16:0)* | **0.48** | **0.34** | **0.41** | **0.46** |
|  |  | 1-stearoyl-GPS (18:0)* | **1.14** | 1.20 | **1.21** | **1.18** |
|  |  | 1-oleoyl-GPS (18:1) | **1.46** | 1.58 | **1.58** | **1.45** |
|  |  | 1-palmitoyl-GPG (16:0)* | **1.87** | **2.04** | **2.22** | **2.16** |
|  |  | 1-stearoyl-GPG (18:0) | **1.23** | **1.42** | **1.33** | **1.28** |
|  |  | 1-oleoyl-GPG (18:1)* | **2.20** | **2.58** | **2.85** | **2.56** |
|  |  | 1-linoleoyl-GPG (18:2)* | **1.66** | **1.73** | **1.67** | **1.74** |
|  |  | 1-palmitoyl-GPI (16:0) | **0.86** | 0.92 | 1.04 | 1.07 |
|  |  | 1-stearoyl-GPI (18:0) | **0.87** | **0.84** | **0.90** | **0.88** |
|  |  | 1-oleoyl-GPI (18:1) | **1.71** | **1.83** | **2.33** | **1.98** |
|  |  | 1-linoleoyl-GPI (18:2)* | **1.07** | **1.28** | **1.28** | **1.22** |
|  |  | 1-arachidonoyl-GPI (20:4)* | **0.59** | **0.55** | **0.59** | **0.66** |
|  | Plasmalogen | 1-(1-enyl-palmitoyl)-2-oleoyl-GPE (P-16:0/18:1)* | **2.30** | **2.45** | **2.59** | **2.14** |
|  |  | 1-(1-enyl-palmitoyl)-2-linoleoyl-GPE (P-16:0/18:2)* | **2.30** | **2.44** | **2.53** | **2.18** |
|  |  | 1-(1-enyl-palmitoyl)-2-palmitoyl-GPC (P-16:0/16:0)* | **0.59** | **0.57** | **0.55** | **0.60** |
|  |  | 1-(1-enyl-palmitoyl)-2-palmitoleoyl-GPC (P-16:0/16:1)* | **3.34** | **4.32** | **3.89** | **3.04** |
|  |  | 1-(1-enyl-palmitoyl)-2-arachidonoyl-GPE (P-16:0/20:4)* | **0.93** | 0.95 | **0.89** | **0.90** |
|  |  | 1-(1-enyl-palmitoyl)-2-oleoyl-GPC (P-16:0/18:1)* | **3.56** | **4.46** | **4.69** | **3.24** |
|  |  | 1-(1-enyl-stearoyl)-2-oleoyl-GPE (P-18:0/18:1) | **1.65** | **1.70** | **1.80** | **1.57** |
|  |  | 1-(1-enyl-stearoyl)-2-arachidonoyl-GPE (P-18:0/20:4)* | **0.62** | **0.56** | **0.58** | **0.62** |
|  | Lysoplasmalogen | 1-(1-enyl-palmitoyl)-GPE (P-16:0)* | **1.96** | **2.00** | **1.90** | **1.63** |
|  |  | 1-(1-enyl-oleoyl)-GPE (P-18:1)* | **2.87** | **3.34** | **3.41** | **2.81** |
|  |  | 1-(1-enyl-stearoyl)-GPE (P-18:0)* | **0.86** | **0.77** | **0.84** | **0.84** |
|  |  | 1-(1-enyl-oleoyl)-2-oleoyl-GPE (P-18:1/18:1)* | **2.84** | **3.13** | **3.42** | **2.65** |
|  | Glycerolipid Metabolism | glycerol | **1.13** | **1.18** | **1.26** | **1.19** |
|  |  | glycerol 3-phosphate | **3.55** | **4.57** | **4.28** | **3.65** |
|  |  | glycerophosphoglycerol | **1.11** | **1.16** | **1.20** | **1.19** |
|  | Monoacylglycerol | 1-myristoylglycerol (14:0) | **2.17** | **2.65** | **2.35** | **2.10** |
|  |  | 1-palmitoylglycerol (16:0) | **2.02** | **2.78** | **2.15** | **1.78** |
|  |  | 1-palmitoleoylglycerol (16:1)* | **4.94** | **8.58** | **7.66** | **6.47** |
|  |  | 1-oleoylglycerol (18:1) | **3.92** | **5.93** | **4.65** | **3.58** |
|  |  | 1-linoleoylglycerol (18:2) | **3.91** | **6.88** | **3.80** | **3.75** |
|  |  | 1-dihomo-linolenylglycerol (20:3) | **5.92** | **11.22** | **7.47** | **5.94** |
|  |  | 1-arachidonylglycerol (20:4) | **6.08** | **8.46** | **7.87** | **8.30** |
|  |  | 1-docosahexaenoylglycerol (22:6) | **1.29** | 1.46 | **1.26** | **1.58** |
|  |  | 2-myristoylglycerol (14:0) | **3.11** | **6.00** | **5.08** | **3.02** |
|  |  | 2-palmitoylglycerol (16:0) | **2.19** | **2.99** | **2.12** | **1.75** |
|  |  | 2-palmitoleoylglycerol (16:1)* | **4.28** | **7.00** | **5.12** | **4.87** |
|  |  | 2-oleoylglycerol (18:1) | **4.47** | **6.56** | **4.53** | **3.79** |
|  |  | 2-linoleoylglycerol (18:2) | **4.52** | **7.89** | **3.95** | **3.92** |
|  |  | 2-arachidonoylglycerol (20:4) | **4.55** | **5.30** | **4.35** | **3.88** |
|  |  | 1-meadoylglycerol (20:3n9)* | **6.37** | **11.01** | **9.00** | **7.60** |
|  | Diacylglycerol | palmitoyl-arachidonoyl-glycerol (16:0/20:4) [2]* | **1.53** | **1.56** | **1.69** | **1.53** |
|  |  | palmitoyl-docosahexaenoyl-glycerol (16:0/22:6) [1]* | **0.31** | **0.24** | **0.27** | **0.33** |
|  |  | stearoyl-arachidonoyl-glycerol (18:0/20:4) [2]* | **1.21** | **1.15** | **1.19** | **1.13** |
|  |  | oleoyl-arachidonoyl-glycerol (18:1/20:4) [2]* | **1.92** | **1.82** | **2.07** | **1.67** |
|  |  | stearoyl-docosahexaenoyl-glycerol (18:0/22:6) [1]* | **0.36** | **0.31** | **0.37** | **0.46** |
|  |  | stearoyl-docosahexaenoyl-glycerol (18:0/22:6) [2]* | **0.45** | **0.37** | **0.43** | **0.50** |
|  | Sphingolipid Synthesis | sphinganine | **0.44** | **0.37** | **0.41** | **0.47** |
|  |  | sphingadienine | **0.88** | **0.84** | **0.77** | **0.85** |
|  |  | phytosphingosine | **0.48** | **0.39** | **0.43** | **0.47** |
|  | Dihydroceramides | N-stearoyl-sphinganine (d18:0/18:0)* | **0.10** | **0.04** | **0.06** | **0.09** |
|  | Ceramides | N-palmitoyl-sphingosine (d18:1/16:0) | **0.29** | **0.22** | **0.22** | **0.26** |
|  |  | N-stearoyl-sphingosine (d18:1/18:0)* | **0.24** | **0.21** | **0.19** | **0.23** |
|  |  | N-palmitoyl-sphingadienine (d18:2/16:0)* | **0.44** | **0.28** | **0.33** | **0.39** |
|  |  | N-stearoyl-sphingadienine (d18:2/18:0)* | **0.39** | **0.29** | **0.32** | **0.37** |
|  |  | N-behenoyl-sphingadienine (d18:2/22:0)* | **0.30** | **0.20** | **0.22** | **0.26** |
|  |  | ceramide (d18:1/14:0, d16:1/16:0)* | **0.36** | **0.20** | **0.23** | **0.25** |
|  |  | ceramide (d18:1/17:0, d17:1/18:0)* | **0.22** | **0.17** | **0.16** | **0.20** |
|  |  | ceramide (d18:1/20:0, d16:1/22:0, d20:1/18:0)* | **0.07** | **0.04** | **0.04** | **0.06** |
|  |  | ceramide (d16:1/24:1, d18:1/22:1)* | **1.74** | **2.18** | **1.93** | **2.11** |
|  | Hexosylceramides (HCER) | glycosyl-N-stearoyl-sphinganine (d18:0/18:0)* | **1.66** | **1.81** | **1.76** | **1.64** |
|  |  | glycosyl-N-palmitoyl-sphingosine (d18:1/16:0) | **2.67** | **3.03** | **3.17** | **2.38** |
|  |  | glycosyl-N-stearoyl-sphingosine (d18:1/18:0) | **2.12** | **2.29** | **2.39** | **2.04** |
|  |  | glycosyl-N-nervonoyl-sphingosine (d18:1/24:1)* | **2.01** | **2.15** | **2.26** | **1.94** |
|  |  | glycosyl-N-(2-hydroxynervonoyl)-sphingosine (d18:1/24:1(2OH))* | **1.05** | 1.00 | **1.10** | **1.11** |
|  |  | glycosyl-N-stearoyl-sphingadienine (d18:2/18:0)* | **3.25** | **3.50** | **3.89** | **2.91** |
|  |  | glycosyl-N-behenoyl-sphingadienine (d18:2/22:0)* | **1.80** | 1.84 | **2.34** | **1.99** |
|  |  | glycosyl ceramide (d16:1/24:1, d18:1/22:1)* | **3.14** | **3.72** | **3.52** | **2.75** |
|  |  | glycosyl ceramide (d18:1/23:1, d17:1/24:1)* | **2.44** | **2.77** | **3.23** | **2.67** |
|  |  | glycosyl ceramide (d18:2/24:1, d18:1/24:2)* | **2.29** | **2.43** | **2.56** | **2.13** |
|  |  | glycosyl ceramide (d18:2/25:1, d18:1/25:2) | **2.01** | **2.02** | **2.15** | **1.86** |
|  | Lactosylceramides (LCER) | lactosyl-N-stearoyl-sphingosine (d18:1/18:0)* | **0.90** | 0.80 | **0.80** | **0.86** |
|  | Glycosphingolipid Sulfates | 3-sulfo-nervonoyl-galactosylceramide (d18:1/24:1) | **2.75** | **3.24** | **3.39** | **2.50** |
|  | Dihydrosphingomyelins | myristoyl dihydrosphingomyelin (d18:0/14:0)* | **1.14** | 0.99 | 1.07 | **1.08** |
|  |  | palmitoyl dihydrosphingomyelin (d18:0/16:0)* | **0.60** | **0.52** | **0.49** | **0.55** |
|  |  | sphingomyelin (d18:0/18:0, d19:0/17:0)* | **0.32** | **0.24** | **0.24** | **0.30** |
|  |  | sphingomyelin (d18:0/20:0, d16:0/22:0)* | **0.14** | **0.07** | **0.08** | **0.13** |
|  | Sphingomyelins | palmitoyl sphingomyelin (d18:1/16:0) | **1.16** | **1.16** | **1.11** | **1.12** |
|  |  | stearoyl sphingomyelin (d18:1/18:0) | **0.61** | **0.54** | **0.56** | **0.61** |
|  |  | behenoyl sphingomyelin (d18:1/22:0)* | **0.63** | **0.63** | **0.55** | **0.55** |
|  |  | tricosanoyl sphingomyelin (d18:1/23:0)* | **1.07** | 1.06 | **1.04** | **1.04** |
|  |  | lignoceroyl sphingomyelin (d18:1/24:0) | **0.75** | 0.76 | **0.65** | **0.66** |
|  |  | sphingomyelin (d18:2/18:1)* | **1.84** | **1.97** | **1.98** | **1.94** |
|  |  | sphingomyelin (d18:2/23:1)* | **3.28** | **3.74** | **4.07** | **3.11** |
|  |  | sphingomyelin (d17:1/14:0, d16:1/15:0)* | **1.79** | **2.21** | **2.02** | **1.93** |
|  |  | sphingomyelin (d18:1/14:0, d16:1/16:0)* | **1.47** | **1.48** | **1.46** | **1.44** |
|  |  | sphingomyelin (d18:2/14:0, d18:1/14:1)* | **1.72** | **1.70** | **1.65** | **1.58** |
|  |  | sphingomyelin (d17:1/16:0, d18:1/15:0, d16:1/17:0)* | **1.41** | **1.46** | **1.38** | **1.37** |
|  |  | sphingomyelin (d18:2/16:0, d18:1/16:1)* | **1.58** | **1.66** | **1.43** | **1.43** |
|  |  | sphingomyelin (d18:1/17:0, d17:1/18:0, d19:1/16:0) | **0.86** | **0.83** | **0.84** | **0.88** |
|  |  | sphingomyelin (d18:1/18:1, d18:2/18:0) | **1.22** | **1.22** | **1.23** | **1.24** |
|  |  | sphingomyelin (d18:1/19:0, d19:1/18:0)* | **0.28** | **0.19** | **0.21** | **0.28** |
|  |  | sphingomyelin (d18:1/20:0, d16:1/22:0)* | **0.25** | **0.18** | **0.19** | **0.25** |
|  |  | sphingomyelin (d18:1/21:0, d17:1/22:0, d16:1/23:0)* | **0.53** | **0.48** | **0.46** | **0.53** |
|  |  | sphingomyelin (d18:1/22:1, d18:2/22:0, d16:1/24:1)* | **2.09** | **2.37** | **2.16** | **1.94** |
|  |  | sphingomyelin (d18:1/22:2, d18:2/22:1, d16:1/24:2)* | **2.87** | **3.84** | **2.76** | **2.70** |
|  |  | sphingomyelin (d18:2/23:0, d18:1/23:1, d17:1/24:1)* | **2.20** | **2.38** | **2.45** | **2.05** |
|  |  | sphingomyelin (d18:1/24:1, d18:2/24:0)* | **1.95** | **2.05** | **2.18** | **1.85** |
|  |  | sphingomyelin (d18:2/24:1, d18:1/24:2)* | **1.89** | **2.09** | **1.85** | **1.69** |
|  | Sphingosines | sphingosine 1-phosphate | **4.87** | **8.17** | **6.13** | **5.29** |
|  |  | hexadecasphingosine (d16:1)* | **0.93** | **0.86** | **0.96** | 0.96 |
|  |  | eicosanoylsphingosine (d20:1)* | **0.34** | **0.24** | **0.27** | **0.32** |
|  | Mevalonate Metabolism | 3-hydroxy-3-methylglutarate | **1.35** | **1.39** | **1.49** | **1.44** |
|  | Sterol | cholesterol | **0.96** | 0.96 | **0.96** | 0.99 |
|  |  | 7-alpha-hydroxy-3-oxo-4-cholestenoate (7-Hoca) | **2.77** | **2.98** | **3.15** | **2.65** |
|  |  | 3beta-hydroxy-5-cholestenoate | **4.07** | **6.71** | **6.31** | **5.37** |
|  |  | 4-cholesten-3-one | **1.52** | **1.23** | **1.56** | **1.30** |
|  |  | 7-hydroxycholesterol (alpha or beta) | **1.48** | **1.46** | **1.53** | **1.46** |
|  | Corticosteroids | corticosterone | **1.32** | **1.23** | **1.26** | **1.31** |
|  |  | cortisol | **1.13** | **1.11** | **1.11** | **1.19** |
|  | Primary Bile Acid Metabolism | glycochenodeoxycholate | **0.85** | 0.95 | **0.77** | 1.06 |
| Nucleotide | Purine Metabolism, (Hypo)Xanthine/Inosine containing | inosine 5'-monophosphate (IMP) | **3.61** | **2.74** | **1.14** | **2.33** |
|  |  | inosine | **1.08** | 1.03 | **1.11** | **1.09** |
|  |  | xanthosine | **1.21** | **1.32** | **1.26** | **1.22** |
|  |  | N1-methylinosine | **1.32** | **1.73** | **1.62** | **1.47** |
|  |  | urate | **0.88** | **0.86** | **0.84** | **0.95** |
|  | Purine Metabolism, Adenine containing | adenosine 5'-monophosphate (AMP) | **2.02** | **1.52** | **1.67** | **1.64** |
|  |  | adenosine 2'-monophosphate (2'-AMP) | **0.88** | **0.66** | **0.69** | **0.81** |
|  |  | adenylosuccinate | **1.88** | **1.64** | **1.37** | **1.29** |
|  |  | adenosine | **0.82** | **0.76** | **0.65** | 0.93 |
|  |  | adenine | **1.19** | **1.21** | **1.15** | **1.17** |
|  |  | N1-methyladenosine | **0.91** | **0.83** | **0.91** | **0.93** |
|  |  | N6-carbamoylthreonyladenosine | **1.13** | **1.13** | **1.15** | **1.21** |
|  | Purine Metabolism, Guanine containing | guanosine 5'- diphosphate (GDP) | **1.38** | **1.36** | **1.44** | **1.39** |
|  |  | guanosine 5'- monophosphate (5'-GMP) | **1.65** | **1.50** | **1.16** | **1.38** |
|  |  | 7-methylguanine | **1.17** | **1.17** | **1.21** | **1.16** |
|  |  | 2'-deoxyguanosine | **0.85** | **0.65** | **0.71** | **0.78** |
|  | Pyrimidine Metabolism, Orotate containing | orotate | **0.85** | **0.79** | **0.82** | **0.91** |
|  |  | orotidine | **1.03** | **1.23** | **1.12** | **1.26** |
|  | Pyrimidine Metabolism, Uracil containing | uridine 5'-monophosphate (UMP) | **2.19** | **1.71** | **1.67** | **1.60** |
|  |  | uridine | **0.93** | **0.84** | **0.93** | **0.95** |
|  |  | uracil | **1.28** | **1.39** | **1.53** | **1.45** |
|  |  | pseudouridine | **0.92** | **0.94** | **0.92** | 0.96 |
|  |  | 5-methyluridine (ribothymidine) | **0.87** | **0.83** | **0.84** | **0.90** |
|  |  | 2'-deoxyuridine | **0.72** | **0.72** | **0.61** | **0.75** |
|  |  | 3-ureidopropionate | **1.03** | 1.07 | **1.18** | **1.12** |
|  |  | N-acetyl-beta-alanine | **0.90** | **0.90** | **0.89** | **1.05** |
|  |  | 3-(3-amino-3-carboxypropyl)uridine* | **0.75** | **0.73** | **0.75** | **0.81** |
|  | Pyrimidine Metabolism, Cytidine containing | cytidine 5'-monophosphate (5'-CMP) | **1.11** | **1.13** | **1.10** | **1.18** |
|  |  | cytidine | **1.35** | **1.60** | **1.42** | **1.57** |
|  |  | 3-methylcytidine | **0.90** | **0.91** | 0.97 | 0.94 |
|  |  | N4-acetylcytidine | **0.87** | **0.86** | **0.83** | 0.90 |
|  | Pyrimidine Metabolism, Thymine containing | thymidine | **0.77** | **0.66** | **0.69** | **0.81** |
|  |  | thymine | **1.21** | **1.34** | **1.30** | **1.33** |
|  |  | 3-aminoisobutyrate | **0.59** | 0.81 | **0.62** | **0.68** |
| Cofactors and Vitamins | Nicotinate and Nicotinamide Metabolism | nicotinamide | **0.89** | **0.90** | **0.90** | **0.92** |
|  |  | nicotinamide ribonucleotide (NMN) | **0.84** | **0.87** | **0.88** | **0.92** |
|  |  | nicotinamide riboside | **0.71** | **0.62** | **0.67** | **0.75** |
|  |  | nicotinamide adenine dinucleotide (NAD+) | **0.58** | **0.54** | **0.51** | **0.61** |
|  |  | nicotinamide adenine dinucleotide reduced (NADH) | **0.59** | **0.50** | **0.56** | **0.62** |
|  |  | 1-methylnicotinamide | **0.90** | **0.79** | **0.88** | **0.86** |
|  |  | trigonelline (N'-methylnicotinate) | **0.90** | 0.82 | **0.86** | **0.91** |
|  |  | N1-Methyl-2-pyridone-5-carboxamide | **0.80** | **0.82** | **0.77** | **0.84** |
|  |  | adenosine 5'-diphosphoribose (ADP-ribose) | **0.88** | **0.73** | **0.84** | **0.89** |
|  |  | NADHX* | **0.66** | **0.52** | **0.61** | **0.68** |
|  | Riboflavin Metabolism | riboflavin (Vitamin B2) | **0.63** | **0.58** | **0.58** | **0.68** |
|  |  | flavin adenine dinucleotide (FAD) | **0.70** | **0.63** | **0.66** | **0.74** |
|  |  | flavin mononucleotide (FMN) | **0.54** | **0.50** | **0.51** | **0.59** |
|  | Pantothenate and CoA Metabolism | pantothenate | **0.92** | **0.85** | **0.88** | **0.92** |
|  |  | pantetheine | **1.15** | 1.10 | **1.30** | **1.36** |
|  |  | phosphopantetheine | **2.14** | **2.74** | **2.47** | **2.38** |
|  |  | 3'-dephosphocoenzyme A | **1.97** | **2.05** | **1.91** | **1.99** |
|  |  | coenzyme A | **2.19** | **2.20** | **2.51** | **2.44** |
|  | Ascorbate and Aldarate Metabolism | ascorbic acid 2-sulfate | **0.68** | **0.84** | **0.72** | **0.87** |
|  |  | dehydroascorbate | **0.80** | **0.69** | **0.76** | **0.78** |
|  |  | 2-O-methylascorbic acid | **1.20** | **1.25** | **1.19** | **1.26** |
|  |  | threonate | **0.82** | **0.75** | **0.79** | **0.82** |
|  | Tocopherol Metabolism | alpha-tocopherol | **1.05** | 1.04 | **1.06** | **1.06** |
|  |  | gamma-tocopherol/beta-tocopherol | **1.27** | **1.36** | **1.28** | **1.21** |
|  | Hemoglobin and Porphyrin Metabolism | heme | **0.40** | 0.50 | **0.30** | **0.36** |
|  |  | biliverdin | **0.59** | **0.49** | **0.49** | **0.46** |
|  | Thiamine Metabolism | thiamin (Vitamin B1) | **0.80** | **0.72** | **0.80** | **0.84** |
|  | Vitamin A Metabolism | retinol (Vitamin A) | **1.43** | **1.50** | **1.45** | **1.36** |
|  |  | carotene diol (1) | **0.62** | **0.50** | **0.57** | **0.60** |
|  | Vitamin B6 Metabolism | pyridoxamine | **0.88** | **0.78** | **0.88** | **0.89** |
|  |  | pyridoxamine phosphate | **0.61** | **0.50** | **0.59** | **0.67** |
|  |  | pyridoxal phosphate | **0.67** | **0.61** | **0.59** | **0.66** |
|  |  | pyridoxal | **0.93** | **0.86** | **0.93** | 0.97 |
| Xenobiotics | Benzoate Metabolism | hippurate | **0.79** | **0.89** | **0.79** | **0.90** |
|  |  | 4-hydroxyhippurate | **0.81** | **0.89** | **0.77** | **0.90** |
|  |  | benzoate | **0.85** | **0.83** | 0.88 | **0.89** |
|  |  | catechol sulfate | **0.65** | 0.92 | **0.76** | 0.94 |
|  |  | 4-methylcatechol sulfate | **0.81** | 0.95 | **0.81** | **0.91** |
|  | Xanthine Metabolism | 3-methylxanthine | **0.78** | **0.86** | **0.74** | **0.89** |
|  | Food Component/Plant | 3-formylindole | **1.18** | **1.19** | **1.29** | 0.99 |
|  |  | gluconate | **0.42** | **0.38** | **0.41** | **0.53** |
|  |  | ergothioneine | **0.85** | **0.77** | **0.81** | **0.89** |
|  |  | erythritol | **0.91** | **0.88** | **0.86** | **0.95** |
|  |  | mannonate* | 0.98 | 0.83 | 0.89 | **1.17** |
|  |  | solanidine | **1.83** | **1.94** | **1.99** | **1.63** |
|  |  | methyl glucopyranoside (alpha + beta) | **1.24** | 1.22 | **1.29** | **1.23** |
|  |  | tartronate (hydroxymalonate) | **0.66** | **0.63** | **0.68** | **0.57** |
|  |  | N-carboxymethylalanine | **1.44** | **1.51** | **1.57** | **1.48** |
|  | Drug - Analgesics, Anesthetics | N-ethylglycinexylidide | **1.13** | 0.98 | 1.08 | 1.22 |
|  | Drug - Cardiovascular | metoprolol acid metabolite* | **0.81** | **0.83** | 1.05 | 0.88 |
|  | Drug - Gastrointestinal | promethazine | **1.32** | 1.03 | 4.47 | 1.20 |
|  | Drug - Neurological | donepezil | **0.73** | **0.67** | **0.72** | **0.78** |
|  | Drug - Topical Agents | hydroquinone sulfate | **0.87** | 0.87 | 0.90 | 0.88 |
|  | Drug - Other | N-carbamoylglutamate | **1.64** | **1.76** | **1.73** | **1.64** |
|  | Chemical | O-sulfo-L-tyrosine | **0.71** | **0.65** | **0.67** | **0.76** |
|  |  | perfluorooctanesulfonate (PFOS) | **1.12** | **1.27** | **1.17** | **1.16** |
|  |  | 4-chlorobenzoic acid | **0.88** | **0.77** | **0.84** | **0.87** |
